# Molecular, Immunological, and Clinical Features Associated With Lymphoid Neogenesis in Muscle Invasive Bladder Cancer

**DOI:** 10.3389/fimmu.2021.793992

**Published:** 2022-01-25

**Authors:** Fabio Pagliarulo, Phil F. Cheng, Laurin Brugger, Nick van Dijk, Michiel van den Heijden, Mitchell P. Levesque, Karina Silina, Maries van den Broek

**Affiliations:** ^1^Institute of Experimental Immunology, University of Zurich, Zurich, Switzerland; ^2^Department of Dermatology, University Hospital Zurich, University of Zurich, Zurich, Switzerland; ^3^Department of Medical Oncology, The Netherlands Cancer Institute, Amsterdam, Netherlands

**Keywords:** tertiary lymphoid structures, lymphoid neogenesis, tumor mutational burden, cancer immunology, immune checkpoint inhibition, survival, prognosis, tumor microenvironment

## Abstract

Lymphoid neogenesis gives rise to tertiary lymphoid structures (TLS) in the periphery of multiple cancer types including muscle invasive bladder cancer (MIBC) where it has positive prognostic and predictive associations. Here, we explored molecular, clinical, and histological data of The Cancer Genome Atlas, as well as the IMvigor210 dataset to study factors associated with TLS development and function in the tumor microenvironment (TME) of MIBC. We also analyzed tumor immune composition including TLS in an independent, retrospective MIBC cohort. We found that the combination of TLS density and tumor mutational burden provides a novel independent prognostic biomarker in MIBC. Gene expression profiles obtained from intratumoral regions that rarely contain TLS in MIBC showed poor correlation with the prognostic TLS density measured in tumor periphery. Tumors with high TLS density showed increased gene signatures as well as infiltration of activated lymphocytes. Intratumoral B-cell and CD8^+^ T-cell co-infiltration was frequent in TLS-high samples, and such regions harbored the highest proportion of PD-1^+^TCF1^+^ progenitor-like T cells, naïve T cells, and activated B cells when compared to regions predominantly infiltrated by either B cells or CD8^+^ T cells alone. We found four TLS maturation subtypes; however, differences in TLS composition appeared to be dictated by the TME and not by the TLS maturation status. Finally, we identified one downregulated and three upregulated non-immune cell-related genes in TME with high TLS density, which may represent candidates for tumor-intrinsic regulation of lymphoid neogenesis. Our study provides novel insights into TLS-associated gene expression and immune contexture of MIBC and indicates towards the relevance of B-cell and CD8^+^ T-cell interactions in anti-tumor immunity within and outside TLS.

## Introduction

In chronically inflamed tissues including cancer, infiltrating lymphocytes can form ectopic lymphoid organs termed tertiary lymphoid structures (TLS) *via* lymphoid neogenesis (1, 2). TLS structurally resemble follicles of secondary lymphoid organs (SLO) like lymph nodes and can exert similar functions such as priming of antigen-specific T cells and mounting ectopic germinal center (GC) reactions (3, 4). TLS density in the tumor microenvironment (TME) correlates with increased infiltration of adaptive immune cells and improved patient survival in a growing list of solid tumors (5, 6) including muscle invasive bladder cancer (MIBC) (7). Along the same lines, TLS density as well as mRNA expression of CXCL13, a crucial regulator of lymphoid neogenesis, has a favorable prognostic association in the context of immune checkpoint inhibition (ICI) therapy of MIBC (8–10). Similar observations were reported in sarcoma, melanoma, and renal cell carcinoma patients (11–13). This has fueled the hypothesis that TLS are drivers of anti-tumor immunity and that, consequently, TLS induction could be considered as a therapeutic strategy (5, 6).

Lymphoid neogenesis and lymphoid organogenesis share central regulatory molecules such as CXCL13, surface lymphotoxin (LTα_1_β_2_), CCL21, and CCL19 (14). During embryogenesis, lymphoid tissue inducer (LTi) cells of hematopoietic origin and lymphoid tissue organizer (LTo) cells of mesenchymal origin interact *via* the abovementioned chemokines and receptor/ligand pairs to establish the developing SLO (15). Such cells are absent in adult organs but various hematopoietic cells like B cells, T cells, and dendritic cells (DC) exhibit functions of LTi cells (16), and stromal cells perform as LTo cells during lymphoid neogenesis (17). Lymphoid organogenesis takes place under sterile conditions, while lymphoid neogenesis requires antigenic stimulation (14). Particularly, T cells and B cells upregulate surface lymphotoxin expression upon activation (18). In peripheral organs, perivascular LTBR-expressing mesenchymal cells respond to surface lymphotoxin-expressing B cells and differentiate into follicular dendritic cells (FDCs), which govern B-cell zone organization and GC activation in TLS as well as in SLO (19–24). In the TME, targeted delivery of LIGHT, an alternative ligand of LTBR produced by activated T cells (25), drives vascular normalization and TLS formation (26, 27). Additionally, the extent of lymphoid neogenesis in a given organ depends also on composition of mesenchymal cells including fibroblasts (14), which affects the overall TLS development in different tumor-bearing organs (28).

Variation in TLS density is observed also between patients of the same tumor type and has significant prognostic associations (5, 6). IL-1R (29, 30) and IFNAR (31) signaling have been implicated as upstream events of lymphoid neogenesis in different inflammatory contexts. Here, we hypothesized that also non-immune, tumor cell-derived factors influence TLS development. To address this, we analyzed MIBC patient data of The Cancer Genome Atlas (TCGA) and the IMvigor210 dataset (32) as well as clinical samples from a retrospective MIBC cohort. Our data demonstrate a significant interaction between TLS density and tumor mutational burden (TMB) as well as increased T-cell activation. A particular feature of TLS-high tumors was the frequent co-infiltration of B cells and CD8^+^ T cells as well as the highest proportion of PD-1^+^TCF1^+^ progenitor-like T cells (33) in intratumoral regions. We found four TLS subtypes based on the presence of FDC and CD8^+^ T cell frequency. As in tumor regions, the highest proportion of PD-1^+^TCF1^+^ progenitor-like CD8^+^ T cells was found in the TLS subtype with the highest B cell and CD8^+^ T cell frequency. However, the main differences in TLS composition were found between TLS from TLS-high and TLS-low tumors rather than between their maturation stages. Finally, we identified one downregulated and three upregulated non-immune-related genes in tumors with high TLS density that are candidates for further studies of tumor-intrinsic regulation of lymphoid neogenesis.

## Materials and Methods

### Patient Cohorts

There are 412 MIBC patient cases available from TCGA. We excluded patients who had no diagnostic images available (*n* = 31) as well as patients for whom there was no peritumoral region visible in the diagnostic image (*n* = 77) (no tumor invasive margin and adjacent organ parenchyma). In total, 304 patients were included in the histological and molecular analyses. From these, 2 patients had no available survival information, 2—no available transcriptomic data and 1—no genomic data, and were thus excluded from the respective analyses. Cohort characteristics are shown in **Supplementary Table 1**.

There are 348 patients available in the IMVigor210 dataset obtained from a multicenter, single-arm, phase 2 trial that investigated efficacy and safety of atezolizumab in metastatic urothelial cancer (32). Clinical response, survival, and gene expression data of these patients were obtained from http://research-pub.gene.com/IMvigor210CoreBiologies/ (34). Cohort details and response criteria were described previously (32). For 50 patients, clinical response was not evaluated, while 25 patients had a complete response, 167—progressive disease, 43—partial response, and 63—stable disease.

A retrospective patient cohort of untreated MIBC patients (*n* = 40) was obtained from the Netherlands Cancer Institute (NKI) (**Supplementary Table 2**). TLS density was assessed in this cohort by mIF. We then selected 12 patients with highest and 11 with lowest TLS density for characterization of TLS-associated tumor immune infiltrates by mIF. Use of retrospective anonymized patient material for research was approved by NKI-AVL institutional research board, following national regulations.

The use of patient material in different analyses of this study is depicted in the study design schematic (**Supplementary Figure 1**).

### TLS Quantification in Histological Images From TCGA

All diagnostic [formalin-fixed paraffin-embedded (FFPE)] and matched cryopreserved sample histology images (H&E staining) of the TCGA MIBC cohort were downloaded through the GDC portal. Dense lymphocytic clusters were marked as TLS annotations using the QuPath software. Presence of GC was also marked in TLS with the characteristic central morphology (**Supplementary Figure 2**). The generated annotations were exported to ImageJ software for automated measurement of TLS and GC number and size as well as total tissue area in each image. In diagnostic images, TLS and GC density was calculated as the number of TLS or GC annotations per measured tissue area. Average TLS or GC size was calculated as the mean area of the TLS or GC annotations for each patient. In cryopreserved sample images, TLS were assessed as present or absent.

### Survival Analysis

Comparison of overall survival across different clinical, histological, and molecular parameters was performed by Kaplan–Meier curve and log-rank test. Patient groups were defined by median cutoffs for all continuous variables. Death was considered as event and patients alive at the last follow-up were censored. Hazard ratio for the different parameters was calculated by univariate Cox regression analysis. Prognostic independence of parameters with significant survival associations in univariate Cox regression was tested by multivariate Cox regression analysis.

### Analysis of Molecular Data

All analysis were performed using R version 4.1.0.

MIBC RNA count, somatic mutation, copy number, and methylation data were downloaded with TCGAbiolinks (35). RNA count data were normalized with edgeR (36) and log2+1 transformed. Genes with less than 1 read per million in less than 10 samples were removed.

Gene expression was compared between TLS density groups. A change in average expression level of 50% (corresponding to fold change of 1.5 and log2 fold change of 0.585) was considered relevant. Two group comparisons were done by two-tailed *t*-test followed by Benjamini and Hochberg adjustment for multiple testing. Genes with adjusted *p*-value <0.05 (corresponding to log10 *p*-value of <−1.3) were considered as differentially expressed. Multiple group comparisons were done by one-way ANOVA.

Pathway analysis of identified differentially expressed genes (DEGs) was done using enrichplot and DOSE (37) for the gene ontology Biological Processes.

Gene signature encompassing the 122 significant TLS-associated DEGs was generated using GSVA (38).

Tumor mutation burden (TMB) was calculated by taking the sum of non-synonymous mutations and insertions and deletions (indels) per patient. Immune cluster and neoantigen data were acquired from Thorsson et al. (39) Abundance of cell population gene signatures was estimated by MCP counter (40).

ImVigor210 data were obtained from http://research-pub.gene.com/IMvigor210CoreBiologies/ (34). RNA count data were normalized with edgeR and log2 +1 transformed, and genes with less than 1 read per million in less than 10 samples were removed. Gene expression was compared between complete responders (*n* = 25) and progressive disease patients (*n* = 167) as described above for TLS density groups.

### TLS–TMB Score

We integrated TLS density and TMB into a joint TLS-TMB score as follows: patients were split into four groups based on the median TLS density (1.08 TLS/cm^2^) and the median mutation count (166 mutations). The categorical score was obtained with 85 patients having both parameters above their medians (HiHi), 86 with both parameters below the medians (LoLo), and 65 patients in each of the mixed groups (HiLo and LoHi, respectively).

The same principle was applied to integrate TLS density and predicted neoantigen load.

Gene expression was compared between TLS-TMB groups (HiHi versus all other) as described above for TLS density groups.

### Immunofluorescence

TLS were analyzed in a retrospective patient cohort by mIF as described before (41). Briefly, 4-μm-thick FFPE tissue sections were treated in Trilogy™ buffer (CellMarque) for 10 min in a pressure cooker at 110°C, blocked in 4% bovine serum albumin/PBS/0.1% Triton X solution, and subjected to sequential detection of CD23 (SP23, Abcam, 1:1,000), CD21 (2G9, Leica, 1:5,000), PNAD (MECA-79, BioLegend, 1:5,000), DC-LAMP (1010E1.01, Dendritics, 1:1,000), CD3 (SP7, ThermoFisher, 1:1,000), and CD20 (L26, Dako, 1:5,000), here referred to as the TLS panel, using tyramide-conjugated fluorophores of the 7-plex Opal system (Akoya). Multispectral images of 200× high-power fields were acquired for all TLS in each patient using the Vectra 3.0 microscope (PerkinElmer/Akoya) with one TLS per image.

For tumor immune infiltrate analysis, tissue processing prior to mIF was done as described above for TLS analysis. The following markers were sequentially detected using a 9-plex Opal system (Akoya): PD-1 (D4W2J, Cell Signaling Technology, 1:8,000), TCF1 (C63D10, Cell Signaling Technology, 1:5,000), CD3 (SP7, ThermoFisher, 1:2,000), CD8 (4B11, Bio-Rad, 1:5,000), CD20 (L26, Dako, 1:5,000), CD21 (2G9, Leica, 1:5,000), Pan-cytokeratin (h-240, Santa Cruz, 1:2,000), and SH2D2A (OTI3C7, ThermoFisher, 1:1,000), here referred to as the TIL panel. A representative set of intratumoral and peritumoral regions as well as all TLS regions in each patient were imaged in high power (200×) using a Vectra Polaris multispectral slide scanner (Akoya). Intratumoral regions were defined as regions not in direct contact with the normal organ parenchyma within the 200× high-power field (**Supplementary Figure 1C**). No intratumoral regions were defined for four patients with highly fragmented tumors that were dispersed throughout the organ parenchyma.

### Quantitative Image Analysis

The workflow of multispectral image analysis of the TLS panel included the following steps: (1) spectral unmixing; (2) tissue segmentation of the following TLS regions: B-cell zone, T-cell zone, follicular dendritic cell (FDC) zone, GC zone, and the remaining non-TLS tissue; (3) measurement of whole TLS area as the sum of the different TLS regions per image; and (4) measurement of total tissue area from the whole slide image. Steps 1–3 were done using the Inform software (version 2.5.1), and step 4 was done using the ImageJ software. TLS maturation stage was determined by the presence or absence of FDC and GC zones in images containing B-cell zones. We did not implement PNAd staining to define TLS area as HEVs are also detected in TLS-non-related regions of the tissue (42). TLS density was calculated as the number of TLS per measured tissue area.

The workflow of multispectral image analysis of the TIL panel (**Supplementary Figure 3**) included the following steps: (1) spectral unmixing; (2) tissue segmentation of tumor, stromal, and TLS regions (B-cell zone, T-cell zone, and FDC zone); (3) measurement of whole TLS area as the sum of the different TLS regions per image; (4) individual cell segmentation; (5) extraction of per-cell fluorescent data, all using the Inform software (version 2.5.1); (6) image data file conversion into flow cytometry data file format using Biobase and flowCore (43); and (7) identification and quantification of different cell phenotypes using flow cytometry data analysis software FlowJo (version 10.8.0) (**Supplementary Figure 3**). The maturation of each TLS was assessed by the presence or absence of the FDC zone by tissue segmentation or by the presence of CD21^+^ cells in single-cell analysis. Tumor, stromal, and TLS tissue segments containing less than 100 cells were excluded from single-cell analysis. Cell frequencies were assessed as the proportion of all cells in each tissue category per image and averaged per patient in peritumoral and intratumoral regions as well as in early and mature TLS.

The immune composition of each tumor and stromal tissue segment was defined based on their frequency of B cells, CD8^+^ T cells, and CD8^-^ T cells, and for TLS composition, CD21^+^ cells were also included. Tissue segments with similar immune composition were identified using hierarchical clustering (R package pheatmap). Frequencies of the identified immune clusters were calculated for each patient as a proportion of the respective tissue segments (tumor, stroma, or TLS).

Cell and cluster frequencies were compared between two groups by two-tailed *t*-test and by one-way ANOVA for multiple group comparisons. No correction for multiple testing was done.

## Results

### TLS Density Is a Significant Positive Prognosticator in Diagnostic Images of MIBC

**Figure 1:**
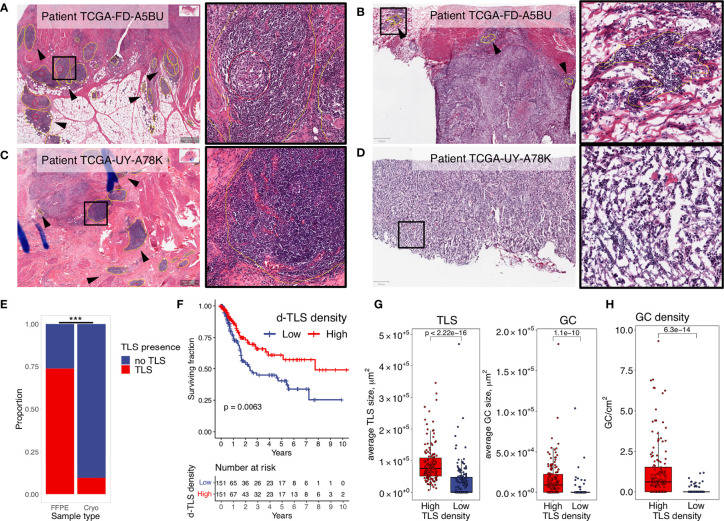
TLS density is a significant positive prognosticator in diagnostic images of MIBC. Dense lymphocytic aggregates were assessed in histology images from diagnostic (FFPE) and matched cryopreserved (Cryo) samples of the TCGA MIBC cohort. TLS (yellow circles) and GC (red circles) were annotated using the Qupath software. Their counts and sizes were measures by ImageJ software. **(A, B)** Representative examples of concordant histology assessment between the two sampling methods: high d-TLS density **(A)** and TLS positivity in the matched cryosample **(B)**. **(C, D)** Representative examples of discordant histology assessment between the two sampling methods: high d-TLS density **(C)** but no TLS in the matched cryosample **(D)**. **(E)** Proportion of TLS-containing images from the diagnostic and cryopreserved samples were compared by Fisher’s exact test (***p < 0.000). **(F)** Median d-TLS density (TLS count per tissue area) was used to define TLS-high and TLS-low patient groups for comparison of overall survival by Kaplan–Meier curve and log-rank test. **(G)** Average size of d-TLS and GC was calculated in each patient and compared between d-TLS density groups by two-tailed *t*-test. **(H)** GC density (count per tissue area) was compared between d-TLS density groups by two-tailed t test.

We quantified TLS in images from diagnostic (FFPE) samples (**Figure 1A**) and matched cryopreserved samples (**Figure 1B**) of the TCGA MIBC cohort (*n* = 304). Cryosample images displayed tissues that were used for the acquisition of TCGA molecular data, and mainly represented intratumoral regions without invasive margin. TLS detected in the cryosamples are here referred to as c-TLS. The diagnostic images always contained intra- and peritumoral regions. We refer to TLS detected in the diagnostic images as d-TLS density (number per tissue area). We found concordance in the histological assessment of TLS between the two sampling methods for approximately 50% of patients: 17 patients with high d-TLS density contained TLS in the matched cryosample (c-TLS-positive) (**Figures 1A, B** and **Supplementary Figure 2A**), and 140 patients with low d-TLS density had no c-TLS. However, a considerable discrepancy was found in 135 cases with high d-TLS density that had no detectable c-TLS (**Figures 1C, D** and **Supplementary Figure 2B**), and in 12 cases with low d-TLS density that had TLS in their cryosample (**Supplementary Figure 2C**). Overall, we detected dense lymphocytic aggregates in approximately 10% of cryosamples (*n* = 29) and in approximately 75% of diagnostic images (*n* = 224) (**Figure 1E**, **Supplementary Figure 2**). Thus, the sensitivity of TLS detection was significantly higher in the diagnostic samples (Fisher’s exact test *p* = 0.000), which may be explained by the fact that intratumoral TLS were considerably less frequent than peritumoral TLS within the same diagnostic image (**Supplementary Figures 2A–E**). Consequently, we used d-TLS density for survival analysis. Patient groups split by median d-TLS density showed a significant difference in overall survival (**Figure 1F**), while groups defined as d-TLS-positive or -negative showed only a trend (**Supplementary Figure 4**), establishing the cohort median as a biologically relevant threshold. We observed that the average size of TLS and GC, as well as the density of GC was positively associated with TLS density (**Figures 1G, H** and **Supplementary Figures 6A–D**), suggesting that tumor microenvironments where TLS are initiated also allow subsequent maturation and generation of GC.

### Intratumoral Gene Expression Poorly Correlates With d-TLS Density

**Figure 2:**
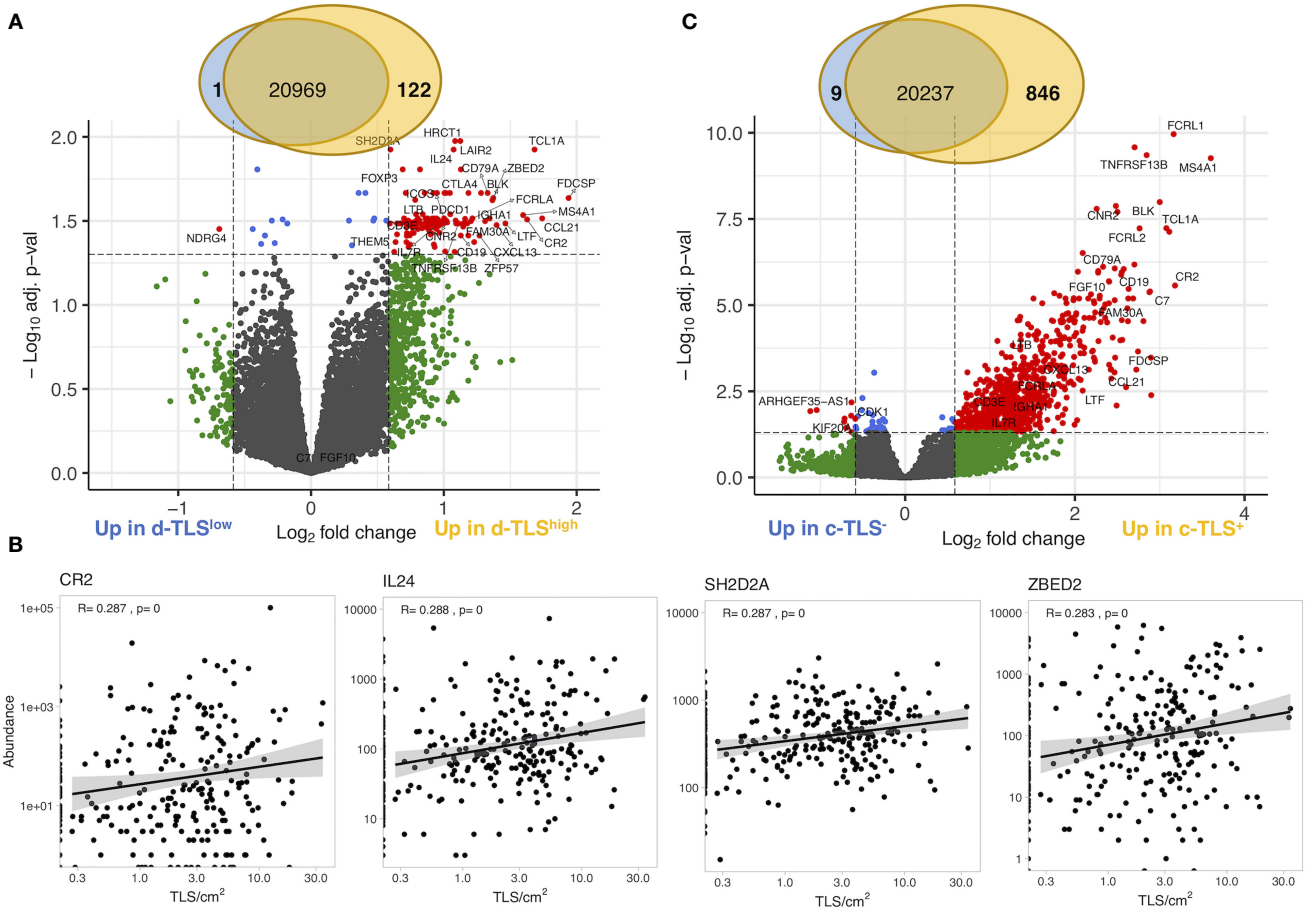
Gene expression in intratumoral regions poorly correlates with d-TLS density. **(A)** Gene expression measured by bulk tumor RNA sequencing of intratumoral regions (cryosamples) was compared in d-TLS density groups by two-tailed *t*-test with Benjamini–Hochberg adjustment for multiple testing. Differentially expressed genes were defined as genes with expression change of 50% (log2 fold change of 0.585) and adjusted *p*-value <0.05 (log10 adj. *p*-value <-1.3) and are displayed as red dots in the Volcano plot. **(B)** Spearman correlation analysis was done to establish the direct relationship between intratumoral DEG abundance and d-TLS density. Representative plots of the most significant correlations are shown. **(C)** Gene expression was compared in patient groups defined by the presence or absence of TLS in their cryosamples (c-TLS) by two-tailed *t*-test and Benjamini–Hochberg adjustment for multiple testing. Significant DEGs were defined and visualized as in **(A)**.

We next investigated whether gene expression profiles obtained from intratumoral regions (cryosamples) could be used as proxies for d-TLS density substituting the necessity of histological evaluation. We thus compared d-TLS-high and -low patient groups and found 123 DEGs (**Figure 2A** and **Supplementary Figure 5A**). Many of these DEGs were canonical TLS-associated genes, like *CR2* (encoding CD21), multiple B cell lineage markers such as *MS4A1* (encoding CD20), *CD19*, and *CD79A*, as well as TLS-relevant chemokines including *CXCL13*, *LTB*, *CCL21*, and *CCL19*. However, we found only weak direct correlations between d-TLS density and the intratumoral mRNA abundance of the DEGs (**Figure 2B** and **Supplementary Table 4**). Similar results were obtained when all TLS-associated DEGs were analyzed as a joint signature (**Supplementary Table 4**), or when immune cell abundance estimated with MCP counter was analyzed (**Supplementary Figures 5B, C**). In fact, most of the identified DEGs showed a much more pronounced expression difference between tumors that did or did not contain c-TLS (**Figure 2C** and **Supplementary Table 5**). Furthermore, we found no association between d-TLS density and the previously published TCGA immune subtypes (immune gene signatures) with prognostic relevance in a pan-cancer setting (39) (**Supplementary Figures 5D, E**). When the MIBC cohort was analyzed separately, the pan-cancer immune subtypes did not show any correlation with survival (**Supplementary Figure 5F**). These results indicate that mRNA profiles obtained from intratumoral regions, which rarely contain TLS (**Supplementary Figure 2D**), cannot be reliably used as proxy for the detection of tumor-associated TLS, which largely develop in the periphery of MIBC (**Supplementary Figure 2D**) and other solid tumors (8, 42, 44).

### Genes Associated With d-TLS Density in the TME Are Dominantly Immune Cell-Related

**Figure 3:**
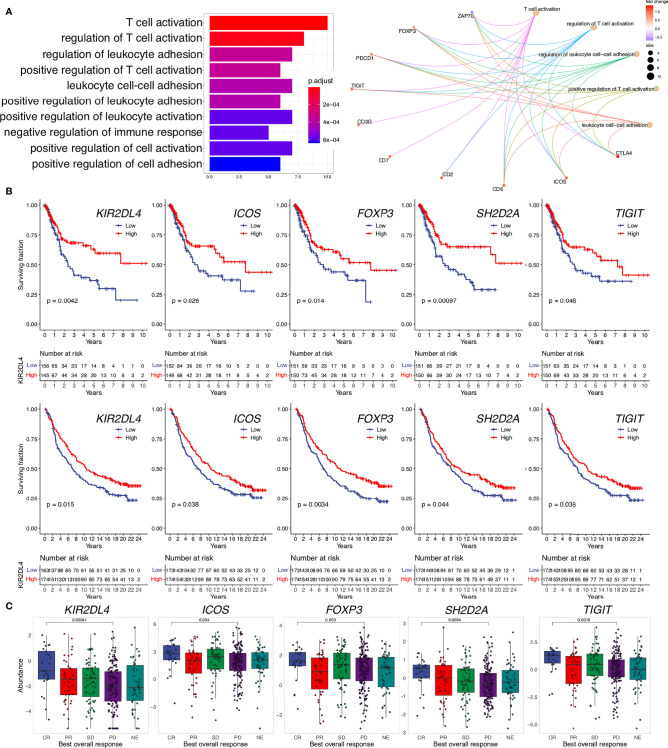
Genes upregulated in TLS-high tumors are related to immune response activation, improved survival, and response to ICI. **(A)** Pathway enrichment analysis of TLS-associated DEGs unique to the d-TLS group comparison (*n* = 44) was performed using gene ontology “Biological Processes”. The number of corresponding DEGs is visualized for the top ten enriched processes (left). The contribution of specific genes to the top five enriched pathways is visualized (right). **(B)** Overall survival was compared for gene expression groups defined by cohort medians in the TCGA MIBC cohort (top row) and IMvigor210 cohort (bottom row) using Kaplan–Meier curve and log-rank test for each unique DEG. **(C)** Expression comparison of the identified unique DEGs between complete response (CR) and progressive disease (PD) after ICI in the IMVigor210 dataset was performed by two-tailed *t*-test. Genes that showed significant favorable prognostic associations in both cohorts are shown.

To search for tumor-intrinsic gene expression alterations with potential impact on lymphoid neogenesis, we first excluded the DEGs that would be derived directly from TLS such as all DEGs overlapping between d-TLS and c-TLS group comparison (**Supplementary Table 5**). As a result, a set of 44 DEGs unique to the d-TLS group comparison remained (**Supplementary Table 6**). Next, we interrogated the published literature and online expression databases to identify potential tumor cell-, immune cell-, and stromal cell-derived genes. Out of the 44 d-TLS-associated genes, 38 were protein coding. Four of those, namely, *NDGR4, ZFP57, THEM5*, and *HRCT1*, did not show an immune cell-associated expression pattern (**Supplementary Table 6**). *NDGR4* was in fact the only gene associated with reduced TLS development in the DEG analysis, while *ZFP57, THEM5*, and *HRCT1* showed increased expression in TLS-high tumors (**Figure 3A**). We found that multiple immune population gene signatures were positively associated with the expression of ZFP57 and HRCT1, and a negative association was found for NDGR4 (**Supplementary Figure 5G**); however, no correlation with patient survival was found for these genes (**Supplementary Table 6**). Furthermore, three genes—*Cxorf65, SSTR3*, and *ART3*—had a testis-associated expression pattern (**Supplementary Table 6**) and might represent novel Cancer/Testis antigens. The majority of the remaining DEGs were, however, associated with activated immune response, in particular, with T-cell activation as demonstrated by pathway enrichment analysis (**Figure 3A**). Expression of most of these genes showed significant correlation with improved patient survival in the TCGA MIBC cohort as well as with response to immune checkpoint inhibition and survival analyzed in the IMVigor210 data set (**Figures 3B, C** and **Supplementary Table 6**). Taken together, TLS in the TME are associated with expression changes of predominantly immune response-related genes; nevertheless, also non-immune genes were found to be altered in TLS density groups and represent candidates for studying tumor-intrinsic regulation of lymphoid neogenesis.

### Joint TLS–TMB Score Is a Novel Independent Prognosticator in MIBC

**Figure 4:**
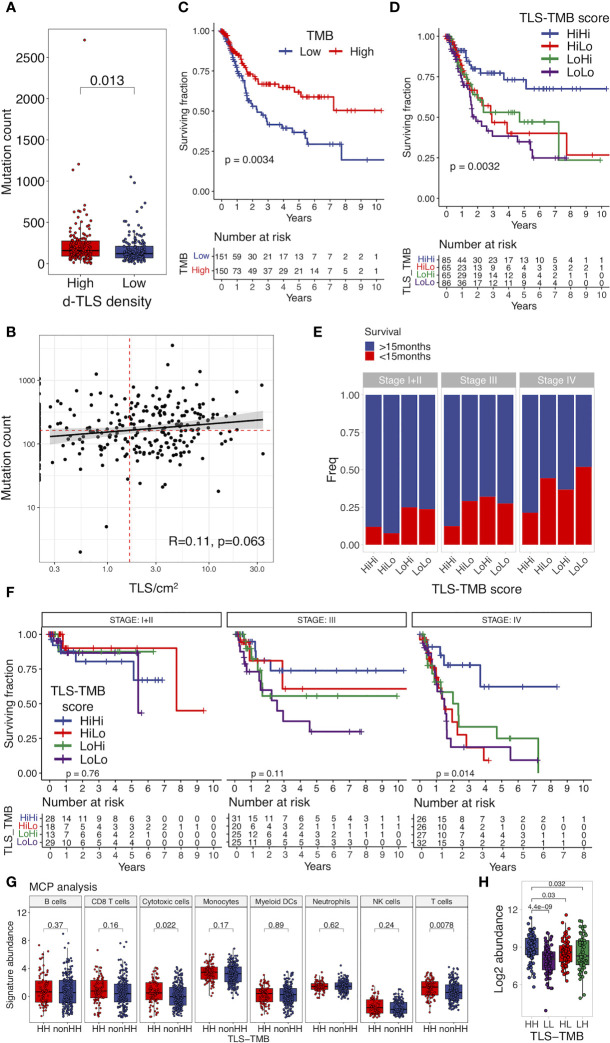
Joint TLS-TMB score is a novel independent prognostic biomarker in MIBC. **(A)** Tumor mutational burden (TMB) was measured as the count of nonsynonymous mutations and was compared in patients with high and low d-TLS densities by two-tailed *t*-test. **(B)** Direct correlation between TMB and d-TLS density was analyzed by Spearman correlation. Red dotted lines represent cohort medians of the TMB and TLS density, respectively. **(C)** Patients with high and low TMB were defined by the cohort median TMB. Overall survival was compared in TMB-high and TMB-low patients by Kaplan–Meier curve and log-rank test. **(D)** MIBC patients were split into four groups based on their high or low d-TLS density in combination with high or low TMB creating a joint TLS-TMB score. Overall survival was compared in the four patient groups by Kaplan–Meier curve and log-rank test. **(E)** Patients were stratified into short-term and long-term survivors by using 15-month survival as cutoff. The proportion of patients with short- and long-term survival in the different TLS-TMB groups was compared in the context of tumor stage. **(F)** Overall survival of different TLS-TMB groups was analyzed in each MIBC stage separately by Kaplan–Meier curve and log-rank test. **(G)** Log2 abundance of cell population gene signatures was determined using the R package MCP counter and compared between TLS-TMB high–high (HH) and other (nonHH) patients by two-tailed *t*-test. **(H)** Gene expression measured by bulk tumor RNA sequencing was compared between TLS-TMB high–high and all other patients by two-tailed *t*-test with Benjamini–Hochberg adjustment for multiple testing. The expression of the only identified protein-coding DEG, SH2D2A, was compared for the TLS-TMB high–high group against all other groups’ two-tailed *t*-test with no adjustment for multiple testing.

We next explored genetic and epigenetic features in MIBC such as copy number variation, methylation profile, TMB, and mutation frequency of individual genes in the context of TLS development. Among these, we found significant differences in TLS density groups only for TMB; the number of nonsynonymous mutations was significantly increased in patients with high d-TLS density (**Figure 4A**). However, there was no direct correlation between TMB and d-TLS density, size, or GC formation (**Figure 4B** and **Supplementary Figures 6A–D)**. Since TMB by itself also conferred favorable prognosis in MICB (**Figure 4C**), we tested whether there is a potential biomarker synergy between TLS and TMB. Indeed, patients with high d-TLS density and high TMB (both parameters above the cohort medians) had a superior overall survival than all other patients (**Figure 4D**). The favorable prognostic interaction was particularly evident in advanced stage patients (**Figures 4E, F**). Neither TLS density nor TMB was associated with tumor stage (**Supplementary Figures 6E, F**). The integrated TLS-TMB score was an independent prognostic marker of survival when tested in a multivariate Cox regression model together with tumor stage and vascular invasion parameters (**Table 1**), while TLS density and TMB as separate parameters showed independence from each other (**Supplementary Table 3**, Model 1) but not from tumor stage or vascular invasion (**Supplementary Table 3**, Model 2). Like TMB, predicted neoantigen count was also increased in patients with high d-TLS density (**Supplementary Figure 3G**). Subsequently, we obtained similar survival associations when TLS density was integrated with predicted neoantigen count (**Supplementary Figure 3H**). The MCP counter analysis revealed that patients with TLS-TMB high–high status had significantly higher expression of cytotoxic cell- or T cell-related gene signatures in comparison to all other patients (**Figure 4G**). We observed similar trends when comparing stage IV patients with high–high versus high–low status (**Supplementary Figure 6I**). Differential gene expression analysis returned a single protein coding gene that was associated with the high–high status—SH2D2A, a T cell-specific adaptor protein with poorly understood functions (45) (**Figure 4H**). Thus, TLS in the context of potentially increased tumor antigenicity (46) provide a prognostic advantage especially for late-stage MIBC patients.

**Table 1:** Multivariate Cox regression analysis.

Parameter	HR	95% Cl		P value
		lower	upper	
Stage III vs Stage I+II	1.47	0.77	2.81	0.24
Stage IV vs stage I+II+III	3.22	1.65	6.28	0.001
Vascular invasion (Yes vs No)	1.77	1.12	2.80	0.014
TLS-TMB score (nonHiHi vs HiHi)	2.14	1.27	3.60	0.004

### TLS-Rich TME Is Associated With Increased Infiltration of Activated Lymphocytes

**Figure 5:**
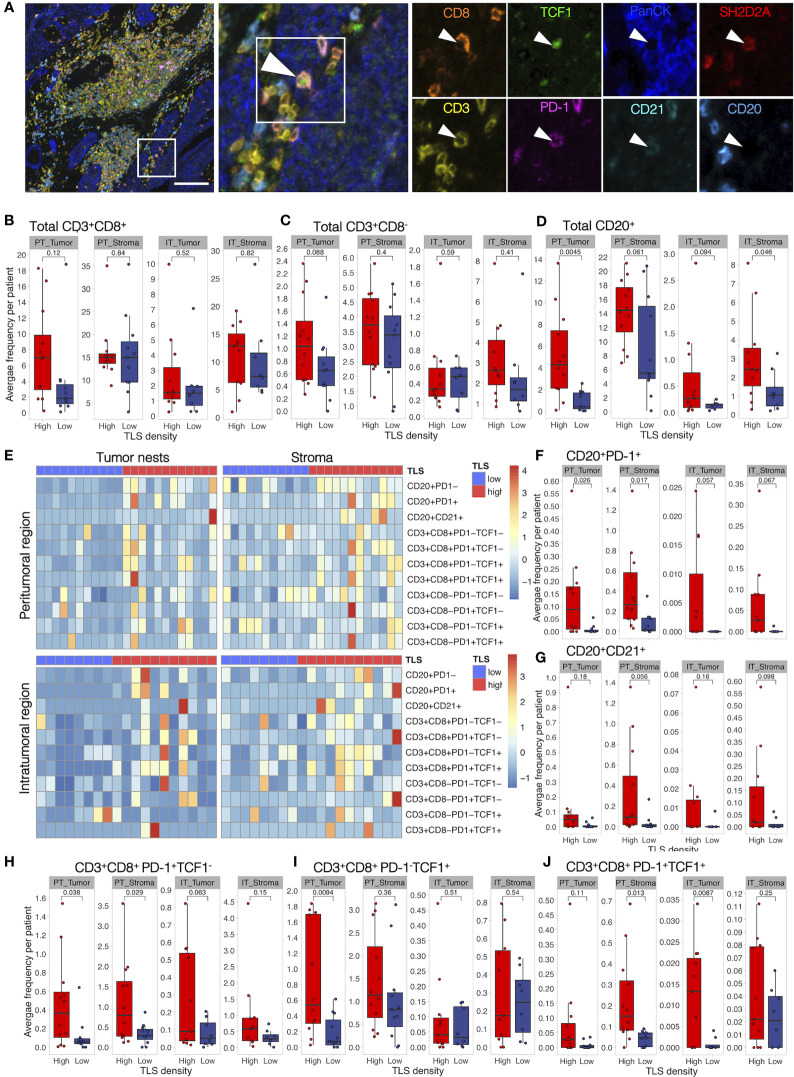
Activated B cells and progenitor-like T cells are increased in TLS-high tumors. Characterization of immune cell phenotypes in TME was performed by mIF staining of CD20 (B cells), CD21 (FDCs), CD3 (total T cells), CD8 (cytotoxic T cells), PD-1 (activation marker), TCF1 (marker of naïve cells), SH2D2A (T cell-specific adaptor protein), and PanCK (tumor cells). Images were acquired by multispectral microscopy from a representative set of peritumoral (PT) and intratumoral (IT) regions. Per-cell fluorescent data were obtained by cell segmentation in tumor nests and stromal regions. **(A)** A representative image of a TLS-high tumor and corresponding mIF staining identifying PD-1^+^TCF1^+^ (progenitor-like) CD8^+^ T cell infiltrating a tumor nest. Scale bar = 100 μm. **(B–D)** Total CD8^+^ **(B)** and CD8^-^ T-cell **(C)** and B-cell **(D)** frequencies were measured as the proportion of all cells within the respective tissue categories (tumor or stroma) and averaged per patient in peritumoral (PT) and intratumoral (IT) regions. Infiltration in the two d-TLS density groups was compared by two-tailed *t*-test. No correction for multiple testing was done. **(E)** Heatmaps displaying z-scaled average frequencies of each measured subpopulation in each tissue category (tumor or stroma) in peritumoral and intratumoral regions for all analyzed patients. **(F–J)** Frequencies of PD-1^+^ **(E)** and CD21^+^ **(G)** B cells, PD-1^+^TCF1^-^ **(H)**, PD-1^-^TCF1^+^ **(I)**, as well as PD-1^+^TCF1^+^ **(J)** CD8^+^ T cells were quantified and compared as described in **(B)**.

We then analyzed tumor immune infiltration in the context of TLS density in a retrospective MIBC cohort. We performed tissue segmentation of 9-plex mIF images to address immune infiltration separately in tumor nests and tumor stroma obtained from peri- and intratumoral regions as well as in TLS (**Supplementary Figure 1C**). To characterize T cells, we selected SH2D2A, which, although with a low fold change, was among the top significant d-TLS-associated DEGs (**Figure 2B**, **Supplementary Figure 5A**, **Supplementary Table 6**), and was uniquely associated with the TLS-TMB high–high status (**Figure 4H**). Additionally, we used PD-1 to detect activated B cells (47–50) and T cells [PD-1^+^ TCF1^-^ (51)], and TCF1 to detect naïve [PD-1^-^ TCF1^+^ (51)] or progenitor-like [PD-1^+^ TCF1^+^ (51)] T cells with known association to improved survival and response to immunotherapy (33) (**Figure 5A** and **Supplementary Figure 3**). In line with a previous report (52), we found that CD8^+^ T cells were the most prominent TIL population in MIBC followed by B cells and CD8^-^ T cells (**Figures 5B-D**). Intratumoral heterogeneity of immune cell infiltration was observed in all patients (**Supplementary Figure 7A**). In line with the variability and average abundance of immune population gene signatures observed by the MCP counter analysis (**Supplementary Figure 5B**), we saw increased average B-cell infiltration and trends towards increased total T-cell frequencies in TLS-high tumors; however, the effect was confined mainly to the peritumoral regions (**Figures 5B-D**). The average frequencies of SH2D2A-expressing cells, PD-1^-^TCF1^-^ CD8^+^ T cells, or all subpopulations of CD8^-^ T cells were not altered in d-TLS density groups (**Supplementary Figures 7B–D**). In contrast, CD8^+^ T-cell and B-cell subpopulations expressing PD-1 were significantly increased in the peritumoral areas of TLS-high patients (**Figures 5E, F, H**). CD21-expresing B cells were found rarely outside mature TLS and showed a trend towards increased infiltration in TLS-high tumors (**Figure 5G**). Furthermore, naïve CD8^+^ T cells (**Figure 5I**) as well as PD-1^-^ B cells (**Supplementary Figure 7C**) were increased in the periphery of TLS-high tumors, while progenitor-like PD-1^+^TCF1^+^ CD8^+^ T cells infiltrated intratumoral regions significantly less in TLS-low patients (**Figure 5J**). Together, TLS are associated with increased infiltration of CD8^+^ T cells and B cells in the TME, recapitulating the result of TCGA gene expression analysis.

### TLS-High Tumors Show Frequent B- and T-Cell Co-Infiltration Harboring Naïve and Progenitor-Like T Cells and Activated B Cells

**Figure 6:**
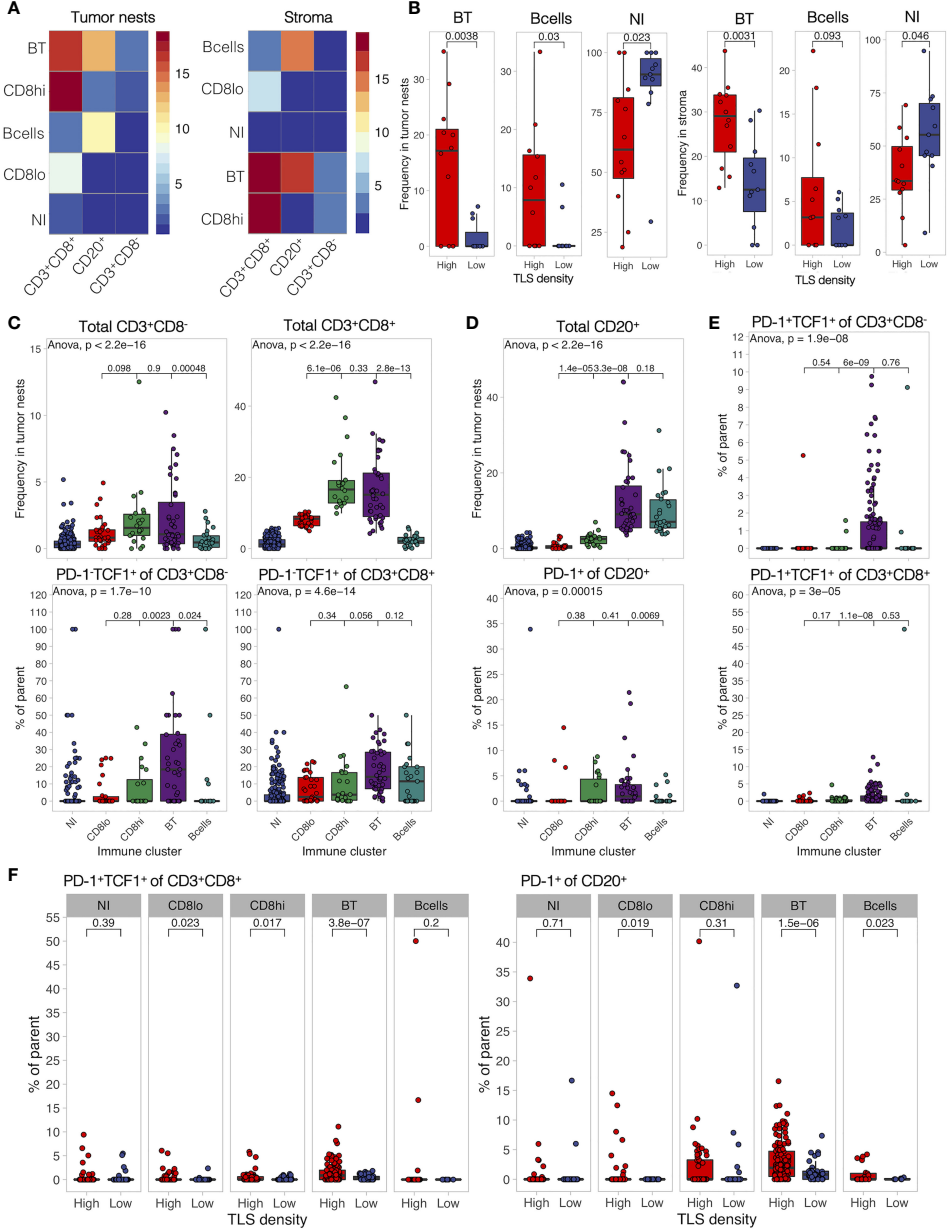
B- and T-cell co-infiltration is common in TLS-high tumors and shows differential immune composition. **(A)** Tumor and stromal regions were classified based on their composition of B cells, CD8^-^, and CD8^+^ T cells by hierarchical clustering. Five immune clusters were identified in both regions: (1) B- and T-cell co-infiltrated (BT, both populations with frequency >5%), (2) B cell-dominant (B cells, B cells >5%, and CD8^+^ T cells <5%), (3) CD8^+^ T cell-dominant high frequency (CD8hi, >10% of cells are CD8^+^ T cells, B cells <5%), (4) CD8^+^ T cell-dominant low frequency (CD8lo, >5% < 10% of cells are CD8^+^ T cells, B cells <5%), and (5) non-infiltrated (NI) (B cells and CD8^+^ T cells both <5%). Heatmaps display the average frequency of the measured cell populations in the identified immune clusters. **(B)** Frequency of each identified immune cluster was determined as the proportion out of all analyzed tumor and stromal segments in each patient. Immune cluster frequencies were compared between TLS-high and TLS-low patients. **(C)** Frequency of infiltrating total CD8^-^ and CD8^+^ T cells (top panels) as well as their proportion of PD-1^-^TCF1^+^ naïve cells (bottom panels) was measured in each individual tissue segment (image) and compared in different tumor immune clusters. **(D)** Frequency of infiltrating total CD20^+^ B cells (top panels) as well as their proportion of PD-1^+^ activated cells (bottom panels) was measured in each individual tissue segment (image) and compared in different stromal immune clusters. **(E)** Proportion of PD-1^+^TCF1^+^ progenitor-like cells from total CD8^-^ (top) and CD8^+^ (bottom) T cells was measured in each tissue category per image and compared in different stromal immune clusters. **(F)** Proportion of PD-1^+^TCF1^+^ progenitor-like CD8^+^ T cells and PD-1^+^ B cells was measured in each individual tissue segment (image) and compared between different TLS density groups for each stromal immune cluster separately. In all panels, two groups (as indicated by group connectors below *p*-values) were compared by two-tailed *t*-test without correction for multiple testing. More than two groups were compared by one-way ANOVA.

To study possible co-infiltration patterns of TILs across different tumor regions, we performed hierarchical clustering of all acquired tumor and stromal tissue segments by their frequencies of B cells, CD8^-^ T cells, and CD8^+^ T cells. We found similar infiltration patterns in tumor nests and stroma, which were classified into five main groups: (1) predominant CD8^+^ T-cell infiltration with high frequency, (2) predominant CD8^+^ T-cell infiltration with low frequency, (3) co-infiltration of CD8^+^ T cells and B cells, (4) predominant infiltration of B cells, and (5) no infiltration (**Figure 6A**). As expected, we found a higher prevalence of non-infiltrated tumor regions in the TLS-low patients, while B-cell dominant and B-cell and CD8^+^ T-cell co-infiltrated regions were more frequent in TLS-high tumors (**Figure 6B**). In fact, B- and T-cell co-infiltration was found in around 20% of tumor regions from 2/3 of the TLS-high patients, which contrasts with less than 10% of tumor regions in 1/4 of TLS-low patients (**Supplementary Figure 8A**). While B- and T-cell co-infiltrated tumor areas had similar total T-cell frequency when compared to T-cell-dominant areas, the proportion of TCF1^+^ T cells (both, CD8^-^ and CD8^+^) was significantly higher (**Figure 6C**). Similarly, PD-1-expressing B cells were enriched in co-infiltrated regions in comparison to B-cell-dominant regions with matching total B-cell infiltration (**Figure 6D**). The same results were obtained for stromal regions (**Supplementary Figure 8B**) that also displayed a significant increase in PD-1^+^TCF1^+^ T-cell proportion (**Figure 6E**). Although the proportion dynamic of the above cell subpopulations in the different immune clusters was similar in both TLS density groups (**Supplementary Figure 8C**), TLS-low tumors exhibited significant reduction in the proportion of these phenotypes compared to TLS-high tumors (**Figure 6F**). Taken together, TLS presence in TME is associated with increased co-infiltration of B cells and CD8^+^ T cells within tumor nests and tumor stroma. Co-infiltrated regions show increased frequencies of naïve (PD-1^-^TCF1^+^) T cells and activated B cells (PD-1^+^) as well as an almost exclusive presence of progenitor-like (PD-1^+^TCF1^+^) T cells when compared to areas predominantly infiltrated by B cells or CD8^+^ T cells alone. These data indicate towards a potential role of B-cell and CD8^+^ T-cell interactions in generating or sustaining PD-1^+^TCF1^+^ T cells.

### Lymphocyte Activation in TLS Is Defined by TME and Not TLS Maturation Stage

**Figure 7:**
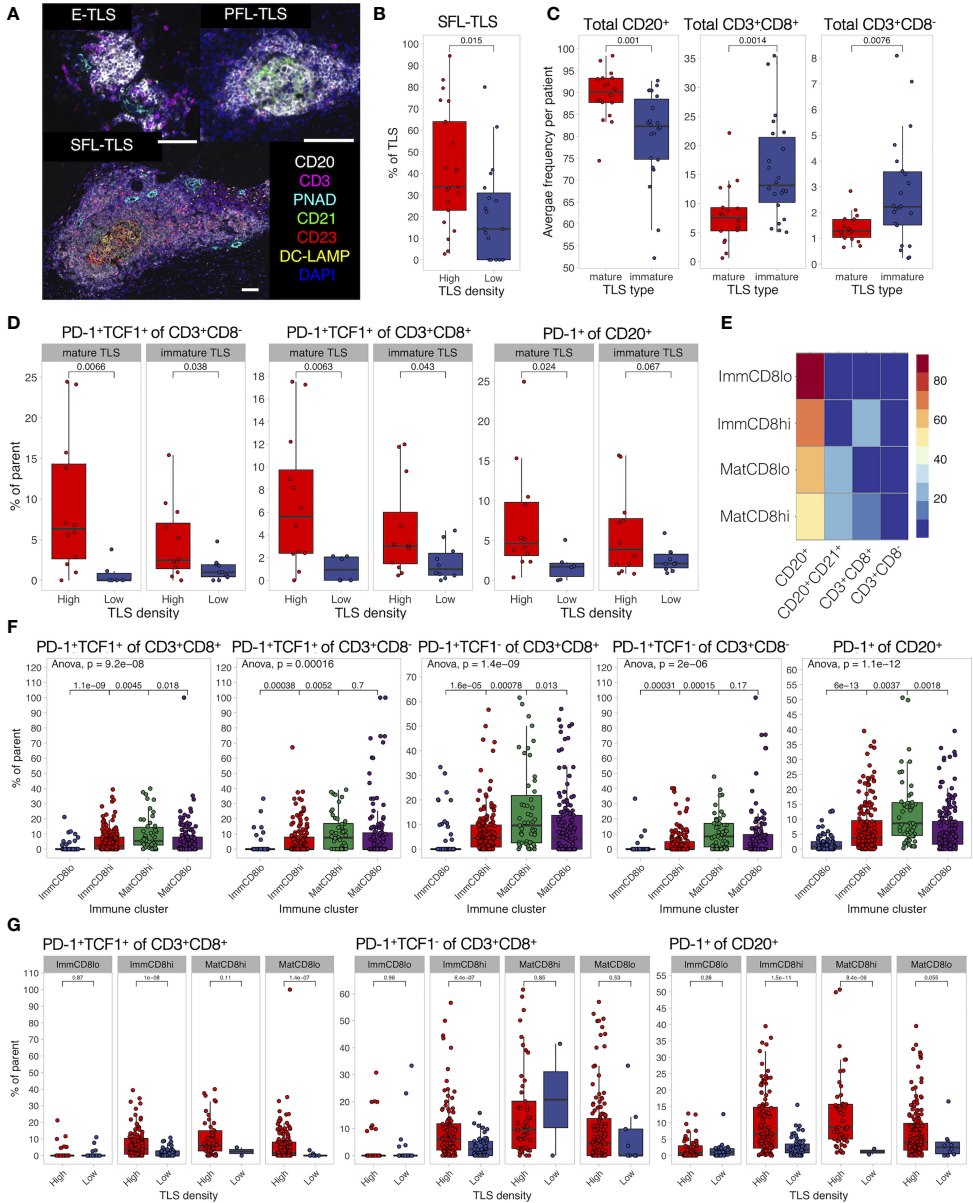
TLS composition depends on TME and differs between maturation clusters. **(A)** TLS quantification and assessment of maturation was performed by mIF staining of CD20 (B cells), CD21 (FDCs), CD3 (total T cells), CD23 (GC cells), PNAd (high endothelial venules), and DC-LAMP (antigen-presenting DCs). Images were acquired by multispectral microscopy from all TLS. Tissue segmentation was used to detect FDC and GC presence in TLS for the assignment of TLS maturation stages as follows: no FDC and GCs—early TLS (E-TLS), FDC networks present but no GC cells—primary follicle-like TLS (PFL-TLS), GC cells present in FDC network—secondary follicle-like TLS (SFL-LS). Representative images of each maturation stage are shown. Scale bar = 100 μm. **(B)** The proportion of each TLS maturation stage was assessed as percentage of total TLS in each patient. The proportion of GC-containing TLS (SFL-TLS) was compared between TLS-high and TLS-low patients. **(C)** Characterization of immune cell infiltration in TLS was performed by mIF staining as described in **Figure 5**. Presence of CD21+ cells was used to define a TLS as mature or immature. Frequencies of B cells and T cells were averaged per patient and compared between TLS maturation stages. **(D)** Proportions of PD-1^+^TCF1^+^ progenitor-like cells from total CD8^-^ (left) and CD8^+^ (center) T cells, as well as PD-1^+^ cells from total B cells (right) were averaged per patient per TLS maturation stage and compared in TLS-high and TLS-low groups. **(E)** Hierarchical clustering was used to identify TLS with similar composition based on B-cell, CD21^+^ B-cell, and T-cell frequencies. Four clusters (TLS subtypes) were identified as follows: immature TLS with high or low CD8^+^ T-cell infiltration (ImmCD8hi and ImmCD8lo, respectively), and mature TLS with high or low CD8^+^ T-cell infiltration (MatCD8hi and MatCD8lo, respectively). Heatmap displays the average frequency of the measured cell populations in the identified TLS subtypes. **(F)** Proportions of PD-1-expressing lymphocytes and PD-1^+^TCF1^+^ progenitor-like T cells were compared in different TLS clusters. **(G)** Proportion of PD-1^+^TCF1^+^ progenitor-like CD8^+^ T cells, PD-1^+^ B cells, and CD8^+^ T cells was measured in each individual TLS and compared between different TLS density groups for each TLS subtype separately. In all panels, two groups (as indicated by group connectors below *p*-values) were compared by two-tailed *t*-test without correction for multiple testing. More than two groups were compared by one-way ANOVA.

Finally, we characterized the development, maturation, and composition of TLS in MIBC patients. We defined TLS stages by the presence of CD21^+^ FDC networks and CD23^+^ GC cells (**Figure 7A**) using image tissue segmentation. Similarly to our previous findings in untreated lung and colorectal cancer (42, 44) as well as the d-TLS assessment in TCGA MIBC cohort (**Figures 2G, H**), TLS were more mature in MIBC patients with high TLS density (**Figure 7B**), suggesting that TLS initiation and maturation in the TME are naturally linked.

Analysis of the cellular composition of TLS by 9-plex mIF revealed that immature TLS had an increased proportion of T cells and reduced proportion of B cells when compared to mature TLS (**Figure 7C**), a feature present in both TLS-high and TLS-low tumors (**Supplementary Figure 9A**). No major differences were seen for the proportions of total B cells and CD8^+^ T cells when each TLS maturation stage was compared in TLS-high and TLS-low tumors separately (**Supplementary Figure 6B**). However, the proportions of PD-1^+^TCF1^+^ T cells and PD-1^+^ B cells in TLS were significantly increased in TLS-high tumors independently of TLS maturation stage (**Figure 7D** and **Supplementary Figure 9C**).

We next analyzed TLS composition following a similar approach as above for tumor and stromal regions. Due to the high cell density within TLS, B cells in contact with FDCs were detected as CD21^+^ and were used here to define the maturation status of TLS subtypes identified by hierarchical clustering analysis (**Figure 7E**). Two mature and two immature TLS clusters were found based on their proportion of CD8^+^ T cells—high and low, respectively, using 10% frequency as the cutoff. As expected, enrichment of mature and immature clusters was observed in TLS-high and TLS-low tumors, respectively (**Supplementary Figures 9D, E**). Interestingly, PD-1^+^TCF1^+^ CD8^+^ T cells, as well as PD-1^+^ B cells and T cells, were highest in the mature-CD8high TLS subtype (**Figure 7F**). However, this effect was only partially preserved when TLS density groups were analyzed separately (**Supplementary Figure 9F**); in TLS-low tumors, these phenotypes were significantly reduced in almost all TLS subtypes (**Figure 7G** and **Supplementary Figure 9G**). To summarize, TLS have reduced T-cell and B-cell activation and progenitor-like T-cell frequency within the TLS-low TME compared to their counterparts in TLS-high TME and independently of maturation stage. Mirroring the findings from the co-infiltrated tumor regions, TLS with high CD8^+^ T-cell and B-cell frequency (such as the mature-CD8high subtype) had the highest proportion of progenitor-like CD8^+^ T cells and PD-1^+^ B cells, which implies the possible role of local interactions between B cells and CD8^+^ T cells in immune response regulation by supporting PD-1^+^TCF1^+^ CD8^+^ T cells.

## Discussion

We have performed comprehensive molecular and histological characterization of MIBC samples in the context of TLS density. We found that TLS formed in the periphery of MIBC in most cases and, in line with previous reports (7, 53), their density correlated with improved survival. We (42) and others [reviewed in (54)] showed that the expression of lymphoid neogenesis-associated chemokines like *CXCL13* and *LTB* as well as lineage markers of TLS components such as B cells is increased in samples containing lymphocytic clusters. Therefore, we reasoned that gene expression signatures could serve as a proxy for TLS quantification in cases where histological material is unavailable. We found that d-TLS density determined by histology of diagnostic sections did not correlate with TLS-related gene expression profiles in intratumoral regions obtained from matched cryosamples. The most likely explanation for this finding is that TCGA transcripts are determined in intratumoral regions that largely lack invasive margins, whereas d-TLS are mostly present around the tumor. The fact that we found a better correlation between TLS-related gene expression and c-TLS positivity supports this explanation. Spatial heterogeneity within tumor samples may further complicate such correlations. Taken together, our data suggest that intratumoral mRNA expression profiles cannot be used as surrogates for adequate TLS assessment in solid tumors with predominantly peritumoral TLS development like lung squamous cell carcinoma (42), CRC (44), and MIBC (8). Consequently, using tumor center-oriented sampling (including TCGA gene expression data or tissue microarray studies) for TLS assessment may not be reliable.

Analysis of genomic alterations revealed that TLS-high tumors were characterized by increased TMB in line with our previous work on colorectal cancer (44). This may be explained by the fact that tumor cells with high TMB potentially contain more neo-antigens and thus better elicit T-cell and B-cell responses (55). In support of this, a study in ovarian and uterine tumors showed that increased TMB was associated with increased infiltration of CD8^+^ T cells expressing *CXCL13* and TLS development (56). Along the same line, higher neoantigen load in pancreatic cancer was observed in patients with increased number of GC-positive TLS and long-term survival (57). Additionally, we found upregulated expression of three testis-associated genes in TLS-high tumors suggesting that these may be candidate Cancer/Testis antigens (58) and further supports the idea of tumor antigenicity as a relevant factor in TLS development. Indeed, a recent report studying an i.p. B16F10 melanoma model showed significantly more spontaneous TLS in ovalbumin-overexpressing than parental tumors, and demonstrated a crucial role of antigen-specific CD8^+^ T cells and activated B cells in the development and maturation of TLS (28).

In our current study, TLS density and TMB did not directly correlate, and were independently associated with patient survival. Combining TLS density with TMB into a joint TLS-TMB score generated a novel prognostic biomarker that, in contrast to either TLS density of TMB alone, was independent from tumor stage and vascular invasion. These results together with previous reports (56, 57) suggest that the improved prognosis of the TLS-TMB high–high patients signifies ongoing anti-tumor immunity. Indeed, increased gene expression related to cytotoxic cells and T cells was found in patients with the TLS-TMB high–high status in comparison to other groups. It will be interesting to investigate the relevance of the joint TLS-TMB score in predicting the clinical response to immunotherapy, especially in the context of PD-1/PD-L1 blockade monotherapies where pre-existing immunity is a relevant biomarker of response (59).

Type I interferons may drive TLS development. In a model of influenza infection, type I interferons induced CXCL13 production in lung fibroblasts, which resulted in the development of TLS (60). We found a potential link between TLS and type I interferons as well: Two genes with known direct involvement in type I IFN signaling—*ZBP1* and *ZBED2—*were upregulated in TLS-high tumors. ZBP1 is a Z-nucleic acid sensing protein that triggers type I IFN production and programmed cell death (61). ZBED2 is a transcriptional repressor of IFN-stimulated genes (62) and has been implicated as a transcription factor typical for antigen-responsive CD8^+^ T cells (63).

Besides type I interferons, we found IL24 as a novel immune-related factor associated with TLS in MIBC. Myofibroblasts and keratinocytes have been reported to produce IL24 in response to inflammatory stimulation, as well as lymphocytes in response to antigen receptor stimulation [reviewed in (64)]. The relevant source of IL24 in the context of TLS development, however, remains to be detemined.

Also, activated T cells correlated with the presence of TLS: Genes related to T-cell activation (*ICOS, FOXP3, SH2D2A*, and others) were among the genes with the highest direct correlation to TLS density. Human *FOXP3* is transiently induced in response to TCR engagement of effector T cells (65); thus, the increased *FOXP3* expression here may signify increased infiltration of activated effector T cells rather than T regulatory cells. In addition, the frequency of activated B cells, T cells, and progenitor-like CD8^+^ T cells was increased in the TME of TLS-high tumors. Furthermore, we observed significantly higher TLS densities as a result of successful ICI in MIBC patients in comparison to non-responders (8).

Previously, we proposed that immature and mature TLS are sequential developmental states in the TME leading to functional TLS (42). Thus, one would anticipate to find increased proportions of activated lymphocytes in TLS with increasing maturation. In contrast to this assumption, however, we found that the proportions of activated B and T cells as well as progenitor-like CD8^+^ T cells were similar when comparing TLS maturation stages defined by the presence of FDCs. The main differences were found instead between TLS from TLS-high and TLS-low TME. Therefore, our results rather suggest that the composition of TLS is orchestrated by the TME. In light of this, we suggest that TLS are a consequence of activated adaptive immunity rather than a prerequisite for the latter. This has important implications for the considerations of potential TLS-targeted therapeutic protocols, suggesting that TLS induction without overcoming the immunosuppressive mechanisms in a TME will not be effective. Furthermore, these results emphasize the importance of in-depth analysis of B- and T-cell zones in the TLS for TLS subtype classification, as suggested by other studies [van Dijk et al., *Frontiers in Immunology*, 2021 (back-to-back submission), and (66)].

We noticed that TLS-high tumors were frequently co-infiltrated by B cells and CD8^+^ T cells. Such tumor regions were enriched with activated B cells, naïve T cells, and progenitor-like T cells, thus mirroring the immune composition of the mature-CD8hi TLS subtype. These data point towards the importance of local B-cell and CD8^+^ T-cell interactions in supporting the progenitor-like T-cell phenotype.

Finally, we identified only a handful of TLS-associated genes that had no known association to immune cells. A single gene was upregulated in TLS-low TME, *NDGR4* (N-Myc downstream-regulated gene 4), which affects cell migration and proliferation. Downregulation of this gene was implicated in promoting breast cancer metastasis (67), although conflicting oncogenic and tumor-suppressive properties have been reported in multiple other tumor types. *HRCT1, THEM5*, and *ZFP57* were upregulated in TLS-high MIBC samples. The functions of HRCT1 are unknown, while THEM5 is required for normal mitochondrial function (68) and ZFP57 is involved in maintaining maternal and paternal gene imprinting (69). These are potential candidate genes for further research on tumor-intrinsic regulation of lymphoid neogenesis.

## Conclusions

We have identified the joint TLS-TMB score as a novel independent biomarker for MIBC patient prognosis. We show that gene expression profiles cannot be extrapolated to TLS density, unless TLS-containing biological samples were used for RNA extraction. This limits TLS analysis to cohorts of solid tumors with available histological images or samples that include peritumoral regions.

We demonstrate that TLS-high tumors are characterized by increased expression of genes related to T-cell activation and contained regions of co-infiltrating B cells and CD8^+^ T cells. Such regions as well as TLS in such tumors hosted increased proportions of progenitor-like CD8^+^ T cells. The composition of TLS seemed to mirror the composition of the TME, and therefore, TLS development may be downstream of an ongoing adaptive immune response.

## Data Availability Statement

The raw data supporting the conclusions of this article will be made available by the authors, without undue reservation.

## Ethics Statement

The studies involving human participants were reviewed and approved by NKI-AVL. The patients/participants provided their written informed consent to participate in this study.

## Author Contributions

KS and MB designed the study. KS wrote the manuscript. FP performed histological assessment of TCGA images. KS and LB performed mIF experiments. PC and ML performed bioinformatics analysis of TCGA molecular data. KS performed the analysis of mIF data and TCGA clinical data. ND and MH provided clinical material and data of the NKI cohort. All authors contributed to the article and approved the submitted version.

## Conflict of Interest

The authors declare that the research was conducted in the absence of any commercial or financial relationships that could be construed as a potential conflict of interest.

## Publisher’s Note

All claims expressed in this article are solely those of the authors and do not necessarily represent those of their affiliated organizations, or those of the publisher, the editors and the reviewers. Any product that may be evaluated in this article, or claim that may be made by its manufacturer, is not guaranteed or endorsed by the publisher.
