# Erratum: Molecular, Immunological, and Clinical Features Associated With Lymphoid Neogenesis in Muscle Invasive Bladder Cancer

**DOI:** 10.3389/fimmu.2022.975797

**Published:** 2022-07-04

**Authors:** Frontiers Production Office

**Affiliations:** Frontiers Media SA, Lausanne, Switzerland

**Keywords:** tertiary lymphoid structures, lymphoid neogenesis, tumor mutational burden, cancer immunology, immune checkpoint inhibition, survival, prognosis, tumor microenvironment

Due to a production error, **Supplementary Figures 2,3,5,6,7,8 and 9** were not published. The publisher apologizes for this mistake.

The original version of this article has been updated.

