# Supplementary material for: Molecular, Immunological, and Clinical Features Associated With Lymphoid Neogenesis in Muscle Invasive Bladder Cancer
Source: Front Immunol. 2022 Jan 25;12:793992. doi: 10.3389/fimmu.2021.793992 (PMC8821902; doi:10.3389/fimmu.2021.793992)
Supplement: Supplementary file 1 [file DataSheet_1.pdf]

Supplemental Table 1. Clinical and histological characteristics of the TCGA MIBC cohort

			Time to Death		
Characteristic	N	n (%)	HR[1]	95% CI[1]	p-value
Stage	303				
I+II		89 (29%)	—	—	
III		102 (34%)	1.94	0.95, 3.94	0.069
IV		112 (37%)	4.16	2.16, 8.03	<0.001
Incidental prostate cancer	249	60 (24%)			
NO			—	—	
YES			1.05	0.63, 1.76	0.84
Histological subtype	299				
Non-Papillary		212 (71%)	—	—	
Papillary		87 (29%)	0.68	0.39, 1.17	0.16
Angiolymphatic invasion	212	112 (53%)	2.90	1.68, 5.02	<0.001
Adjuvant treatment	192	56 (29%)	0.92	0.53, 1.60	0.76
Neoadjuvant treatment	304	9 (3.0%)			
No			—	—	
Yes			1.05	0.26, 4.28	0.95
Gender	304				
FEMALE		80 (26%)	—	—	
MALE		224 (74%)	1.08	0.67, 1.74	0.74
TLS density	304	152 (50%)	0.55	0.35, 0.85	0.007
Germinal centres	304	114 (38%)	0.76	0.49, 1.19	0.23
TMB	303	150 (50%)	0.53	0.34, 0.82	0.004
TLS-TMB score	303				
HiHi		85 (28%)	—	—	
Other		218 (72%)	2.56	1.44, 4.56	0.001

[1] HR = Hazard Ratio, CI = Confidence Interval
