# Supplementary material for: Molecular, Immunological, and Clinical Features Associated With Lymphoid Neogenesis in Muscle Invasive Bladder Cancer
Source: Front Immunol. 2022 Jan 25;12:793992. doi: 10.3389/fimmu.2021.793992 (PMC8821902; doi:10.3389/fimmu.2021.793992)
Supplement: Supplementary file 2 [file DataSheet_2.pdf]

Supplementary Table 2. Clinical and histological characteristics of the NKI MIBC cohort

			Time to Death			
Characteristic	N	n (%)	N	HR[1]	95% CI[1]	p-value
Age	40	62 (57, 70)	40	0.97	0.93, 1.01	0.12
Gender	40	10 (25%)	40	2.05	0.86, 4.92	0.11
pT	40		40	1.81	1.06, 3.09	0.030
2		12 (30%)				
3		10 (25%)				
4		18 (45%)				
pN invasion	40		40			
N0		25 (62%)		—	—	
N1		4 (10%)		0.58	0.08, 4.50	0.61
N2		11 (28%)		4.74	1.83, 12.3	0.001
Adjuvant treatment	40	9 (22%)	40			
No				—	—	
Yes				0.71	0.26, 1.98	0.52
TLS density	40	21 (52%)	40	0.98	0.42, 2.27	0.96

[1] HR = Hazard Ratio, CI = Confidence Interval
