# Supplementary material for: Molecular, Immunological, and Clinical Features Associated With Lymphoid Neogenesis in Muscle Invasive Bladder Cancer
Source: Front Immunol. 2022 Jan 25;12:793992. doi: 10.3389/fimmu.2021.793992 (PMC8821902; doi:10.3389/fimmu.2021.793992)
Supplement: Supplementary file 3 [file DataSheet_3.pdf]

Supplementary Table 3. Multivariate Cox regression analysis

Model 1	HR	95% CI lower	95% CI upper	P-value
TLS density (High vs Low)	0.6005	0.3836	0.9398	0.0256
TMB (High vs Low)	0.5752	0.3699	0.8945	0.0141

Model 2	HR	95% CI lower	95% CI upper	P-value
Stage III vs Stage I+II	1.5296	0.6349	3.685	0.34349
Stage IV vs Stage I+II+III	3.9783	1.6443	9.625	0.00219
Vascular invasion (Yes vs No)	1.9154	1.0621	3.454	0.03075
TLS density (High vs Low)	0.8096	0.4659	0.4659	1.407
TMB (High vs Low)	0.6108	0.3582	1.041	0.07011
