# Supplementary material for: Molecular, Immunological, and Clinical Features Associated With Lymphoid Neogenesis in Muscle Invasive Bladder Cancer
Source: Front Immunol. 2022 Jan 25;12:793992. doi: 10.3389/fimmu.2021.793992 (PMC8821902; doi:10.3389/fimmu.2021.793992)
Supplement: Supplementary file 4 [file DataSheet_4.pdf]

Supplementary Table 4. Spearman correlation analysis between d-TLS density and expression of identified TLS-associated DEGs

Gene	Spearman R	p value
IL24	0.28844449	3.24E-07
CR2	0.28671834	3.83E-07
SH2D2A	0.28668749	3.84E-07
**122genesignature**	0.285	3.84E-07
ZBED2	0.28299554	5.49E-07
FCRLA	0.27654539	1.01E-06
FDCSP	0.27382158	1.30E-06
SSTR3	0.26838812	2.14E-06
LAIR2	0.26785135	2.25E-06
IL22RA2	0.26777981	2.26E-06
ZC3H12D	0.26489078	2.93E-06
FOXP3	0.26368469	3.26E-06
HRCT1	0.26072707	4.24E-06
MYO1G	0.25849666	5.15E-06
FCRL3	0.25759016	5.57E-06
LTF	0.25699061	5.86E-06
TCL1A	0.2566479	6.04E-06
RHOH	0.25645447	6.14E-06
KLHDC7B.DT	0.25578563	6.51E-06
SLAMF1	0.25320206	8.11E-06
NCF1C	0.25286745	8.35E-06
CD79A	0.25127222	9.55E-06
CNR2	0.25118279	9.63E-06
CTLA4	0.25079578	9.95E-06
MS4A1	0.25003749	1.06E-05
LINC01215	0.24942167	1.12E-05
ICOS	0.24492247	1.62E-05
CD6	0.24489562	1.62E-05
CXCL10	0.24323193	1.86E-05
HNRNPA1P21	0.24202091	2.05E-05
CD27	0.2416122	2.12E-05
TYMP	0.23916187	2.59E-05
U62317.3	0.23915119	2.59E-05
CCL21	0.23855848	2.72E-05
CD3D	0.23830416	2.77E-05
MMP12	0.23750557	2.95E-05
FCAMR	0.23712602	3.04E-05
AC004847.1	0.23660581	3.17E-05
SHISAL2A	0.23655046	3.19E-05
LTB	0.23570452	3.41E-05
SLA	0.23552684	3.45E-05
FUT7	0.23470089	3.69E-05
CYTIP	0.23461247	3.71E-05
SCML4	0.23411677	3.86E-05
TRBV5.1	0.23335132	4.10E-05
TRBV7.9	0.23289309	4.25E-05
CXCL13	0.2327601	4.29E-05
IL7R	0.23230488	4.45E-05
MAP4K1	0.23228916	4.45E-05
TRAC	0.23218995	4.49E-05
TRBV28	0.23188439	4.59E-05
CD3E	0.2314565	4.75E-05
CD2	0.23142744	4.76E-05
BLK	0.23138405	4.78E-05
AC007384.1	0.23058252	5.08E-05
NCF1	0.23024764	5.21E-05
TNFRSF9	0.22990162	5.35E-05
ADAMDEC1	0.2294331	5.55E-05
ITK	0.22913159	5.68E-05
PDCD1	0.22911815	5.69E-05
TIGIT	0.22834366	6.03E-05
SAMSN1	0.22790525	6.24E-05
CD7	0.22771128	6.33E-05
TIFAB	0.22638373	7.01E-05

IL21R	0.22625301	7.08E-05
CD22	0.22574879	7.35E-05
CXCR6	0.2251451	7.69E-05
FAM30A	0.22496665	7.80E-05
CD37	0.22325706	8.86E-05
CD3G	0.22320204	8.90E-05
ARHGAP9	0.22266816	9.26E-05
LINC00402	0.22180383	9.88E-05
SASH3	0.22176856	9.90E-05
ADGRG5	0.22107014	1.04E-04
CLEC4E	0.22087821	1.06E-04
ZBP1	0.22025158	1.11E-04
IL2RB	0.22007237	1.12E-04
KIR2DL4	0.21996675	1.13E-04
JCHAIN	0.21990168	1.14E-04
SH2D1A	0.2192413	1.19E-04
FCRL5	0.21922079	1.20E-04
TBC1D10C	0.21891206	1.22E-04
IL16	0.21877436	1.24E-04
TNFRSF13B	0.21798514	1.31E-04
CD19	0.21787345	1.32E-04
BIRC3	0.21755748	1.35E-04
IGLC2	0.21689135	1.42E-04
MZB1	0.21660614	1.45E-04
CHRM3.AS2	0.21604155	1.51E-04
PYHIN1	0.21602391	1.51E-04
P2RY10	0.21596881	1.51E-04
FCMR	0.21575307	1.54E-04
TRAF3IP3	0.21411774	1.73E-04
TNFRSF17	0.21402942	1.74E-04
TRG.AS1	0.21388671	1.76E-04
GPR15	0.21364117	1.79E-04
IRF4	0.2135947	1.80E-04
SLAMF6	0.21186663	2.03E-04
SPN	0.21154418	2.08E-04
SP140	0.21040505	2.25E-04
IGKV1.5	0.21029925	2.27E-04
TRGC2	0.21022964	2.28E-04
JAML	0.209556	2.39E-04
AC243960.1	0.20761539	2.74E-04
CORO1A	0.20719332	2.82E-04
ZNF831	0.20682404	2.89E-04
ITGAL	0.20564499	3.14E-04
LINC02397	0.20506237	3.27E-04
CXorf65	0.20405955	3.50E-04
ZFP57	0.20054938	4.44E-04
TRBV20.1	0.20008514	4.58E-04
LY9	0.19875771	5.01E-04
ART3	0.19869394	5.03E-04
ZAP70	0.1984972	5.10E-04
CFP	0.19277533	7.42E-04
CD5	0.1922787	7.67E-04
FCER2	0.19119928	8.22E-04
TRGC1	0.19082483	8.42E-04
TNFSF8	0.19073225	8.47E-04
LINC00861	0.19048271	8.60E-04
IGHA1	0.187115	0.00106571
THEM5	0.18503627	0.00121396
RIPOR2	0.17280546	0.00254187
NDRG4	-0.1343524	0.01930399
