# Supplementary material for: Molecular, Immunological, and Clinical Features Associated With Lymphoid Neogenesis in Muscle Invasive Bladder Cancer
Source: Front Immunol. 2022 Jan 25;12:793992. doi: 10.3389/fimmu.2021.793992 (PMC8821902; doi:10.3389/fimmu.2021.793992)
Supplement: Supplementary file 5 [file DataSheet_5.pdf]

Supplementary Table 5. Expression of TLS-derived genes in d-TLS and c-TLS group comparisons

	d-TLS^{high} vs d-TLS^{low}		c-TLS^{+} vs c-TLS^{-}	
**Gene name**	**logFC d-TLS**	**adj.P.Val d-TLS**	**logFC c-TLS**	**adj.P.Val c-TLS**
AC007384.1	0.872	0.030541948	1.178287882	0.014966521
AC243960.1	0.766	0.034498733	1.305786655	0.001874005
ARHGAP9	0.717	0.034498733	1.023727984	0.012935631
BLK	1.372	0.023096175	2.999993666	1.02E-08
CCL21	1.740	0.030541948	2.467032641	8.94E-04
CD19	1.128	0.038640763	2.553428209	1.11E-06
CD22	0.963	0.032736882	2.049597913	6.42E-06
CD27	0.841	0.02886061	1.28040993	0.002435367
CD37	0.718	0.042243638	1.358567692	1.55E-04
CD3D	0.914	0.030541948	1.059954062	0.046449536
CD3E	0.868	0.032736882	1.187171449	0.011701773
CD5	0.763	0.034498733	1.26094074	0.002592644
CD79A	1.330	0.021558444	2.469643593	8.59E-07
CFP	0.647	0.032736882	1.145721638	0.001353552
CNR2	1.025	0.032274422	2.482673466	1.33E-08
CORO1A	0.593	0.032736882	0.889651192	0.014731472
CR2	1.627	0.030977192	3.17973621	2.71E-06
CXCL13	1.397	0.033475531	2.044055924	0.001361107
CYTIP	0.805	0.030541948	1.075907191	0.011830381
FAM30A	1.176	0.032736882	2.399980319	8.86E-06
FCAMR	1.203	0.031710633	2.065250428	2.49E-04
FCER2	1.185	0.038640763	2.700603153	6.64E-07
FCMR	0.647	0.037999581	1.265996339	1.49E-04
FCRL3	1.160	0.030732204	2.403375987	2.09E-06
FCRL5	1.228	0.042243638	2.163899175	7.47E-04
FCRLA	1.215	0.030541948	1.84277643	0.00228431
FDCSP	1.939	0.023096175	2.723915218	7.47E-04
FUT7	0.873	0.030977192	1.092153216	0.033909982
GPR15	1.089	0.031477536	1.441356736	0.019633541
IGHA1	1.216	0.030541948	1.627269904	0.00467396
IGKV1-5	1.092	0.032736882	1.466208482	0.013860513
IGLC2	0.923	0.032736882	1.180261585	0.040358405
IL16	0.626	0.048333874	1.192975806	3.23E-04
IL7R	0.925	0.034498733	1.270965185	0.012336134
IRF4	0.867	0.034498733	1.417110711	0.003228812
ITGAL	0.776	0.033704908	1.062142393	0.016236913
ITK	0.927	0.030541948	1.424335226	0.003080852
JAML	0.696	0.032274422	1.182940337	7.49E-04
JCHAIN	1.311	0.031710633	1.802367561	0.00765034
LINC00861	0.896	0.033475531	1.598784605	6.72E-04
LINC01215	0.976	0.032736882	1.357613735	0.013893152
LINC01857	0.931	0.04518225	1.87511346	8.53E-05
LINC02397	0.945	0.035349762	1.959369602	2.34E-05
LTB	0.878	0.030541948	1.523113663	7.45E-05
LTF	1.463	0.032736882	2.091790821	0.003026717
LY9	0.895	0.032736882	1.669027464	2.81E-04
MAP4K1	0.713	0.021558444	0.929160263	0.009869753
MS4A1	1.597	0.029184872	3.601907013	5.45E-10
MYO1G	0.790	0.02886061	0.976351798	0.021426495
MZB1	1.146	0.034202387	1.64006358	0.005608011
NCF1C	0.861	0.03059617	1.27325356	0.006627338
P2RY10	0.908	0.037368376	1.648402454	6.34E-04
PYHIN1	0.861	0.034202387	1.125713742	0.04134723
RHOH	0.921	0.021558444	1.404069238	0.001178413
RIPOR2	0.670	0.032736882	1.038963171	0.00517162
SAMSN1	0.764	0.032736882	1.019021863	0.025045432
SASH3	0.744	0.043666502	1.21873843	0.002348739
SCML4	0.955	0.030977192	1.369536787	0.007767049
SH2D1A	0.943	0.032736882	1.448608019	0.005674541
SLA	0.764	0.032274422	1.054721051	0.012633195
SLAMF1	1.005	0.021558444	1.348238853	0.006654395
SLAMF6	0.972	0.031842882	1.5159603	0.003428206
SPIB	1.081	0.048307882	1.979210726	5.58E-04
SPN	0.721	0.038780516	0.937311433	0.045855558
TBC1D10C	0.733	0.031477536	1.096400994	0.004654983
TCL1A	1.683	0.011885533	3.078105032	5.85E-08
TIFAB	0.969	0.037267715	1.325569127	0.023849867
TNFRSF13B	1.110	0.032736882	2.845266164	4.45E-10
TNFRSF17	1.146	0.032736882	1.587852467	0.013739601
TNFSF8	0.799	0.032736882	1.365769204	0.001786114
TRAC	0.899	0.032147154	1.193809344	0.012932222
TRAF3IP3	0.701	0.030541948	1.033466997	0.005479122
TRBV20-1	0.922	0.032736882	1.367361491	0.007545772
TRBV28	0.921	0.031477536	1.324871342	0.008045264
TRBV5-1	1.128	0.015609525	1.470519681	0.00496852
TRBV7-9	0.964	0.033704908	1.407881468	0.010494376
ZC3H12D	0.820	0.015609525	1.146227829	0.003228812
ZNF831	0.944	0.030541948	1.547189946	0.001172653
