# Supplementary material for: Molecular, Immunological, and Clinical Features Associated With Lymphoid Neogenesis in Muscle Invasive Bladder Cancer
Source: Front Immunol. 2022 Jan 25;12:793992. doi: 10.3389/fimmu.2021.793992 (PMC8821902; doi:10.3389/fimmu.2021.793992)
Supplement: Supplementary file 6 [file DataSheet_6.pdf]

Supplementary Table 6. Characteristics of DEGs found uniquely in d-TLS group comparison

Gene name	Expression in TLS-high versus TLS-low TCGA MIBC samples		Function or process	Expression pattern from Proteinatlas.org, NCBI Gene, gtexportal.org or Genecards.org	Gene type	Survival associations in urothelial cancer from Proteinaltas.org	Univariate COX regression on TCGA cohort (as continuos variables)				Expression in IMvogor210 complete responders vs progressive disease	
	log2FC	adj.P.Val					beta	HR (95% CI)	Wald test	p.value	log2FC6	P.Value7
AC004847.1	0.818	0.031	lncRNA, unknown	multiple tissues	non protein coding	NA	-0.0078	0.99 (0.99-1)	5.3	0.021	1.307	0.000
ART3	0.902	0.038	modification of proteins by adding or removing ADP-ribose	Testis, muscle	testis-associated	improved prognosis	-0.0027	1 (0.99-1)	1.2	0.26	0.045	0.907
BIRC3	0.806	0.032	Apoptosis, regulates NFkB cascade, modulates inflammation	T cells, B cells, granulocytes	immune-related	improved prognosis	-0.00016	1 (1-1)	5.3	0.021	0.469	0.135
CD2	0.906	0.033	adhesion and costimulatory receptor	T cells, NK cells	immune-related	improved prognosis	-0.00048	1 (1-1)	6.2	0.012	0.825	0.011
CD3G	0.734	0.045	activation of downstream pathway upon TCR activation	T cells	immune-related	improved prognosis	-0.0019	1 (1-1)	6.1	0.014	0.919	0.002
CD6	0.855	0.022	cell-adhesion, T cell activation	T cells	immune-related	not prognostic	-0.00041	1 (1-1)	3	0.083	0.816	0.005
CD7	0.865	0.032	regulates adhesion and apoptosis	T cells, NK cells	immune-related	improved prognosis	-0.00065	1 (1-1)	7.7	0.0056	0.838	0.010
CLEC4E	0.959	0.033	pattern recognition receptor	Myeloid cells	immune-related	not prognostic	-0.00089	1 (1-1)	1.1	0.29	0.600	0.114
CTLA4	1.017	0.022	immune checkpoint	T cells	immune-related	improved prognosis	-0.0017	1 (1-1)	6.4	0.011	0.807	0.028
CXCR6	0.722	0.037	chemokine receptor, T cell migration in peripheral tissues	T cells	immune-related	improved prognosis	-0.0015	1 (1-1)	8.4	0.0039	0.779	0.011
CXorf65	0.773	0.037	unknown	Predominantly testis	testis-associated	improved prognosis	-0.015	0.99 (0.97-1)	4	0.044	0.632	0.076
FOXP3	0.690	0.016	transcription factor	T cells	immune-related	not prognostic	-0.0012	1 (1-1)	4.7	0.031	0.460	0.082
HRCT1	1.085	0.011	unknown	multiple tissues	nonimmune	not prognostic	-0.0004	1 (1-1)	0.19	0.66	-0.874	0.010
ICOS	1.002	0.031	co-stimulatory molecule	T cells	immune-related	improved prognosis	-0.003	1 (0.99-1)	7.3	0.0069	0.751	0.032
IL21R	0.888	0.036	promotion of lymphocyte proliferation and differentiation	NK cells, B cells, T cells	immune-related	not prognostic	-0.00046	1 (1-1)	1.9	0.17	0.731	0.015
IL22RA2	1.185	0.022	secreted receptor, inhibits IL22 activity	Syncytiotrophoblast (placenta), B cells	immune-related	improved prognosis in advanced stage	-0.00064	1 (1-1)	0.27	0.6	0.303	0.493
IL24	1.074	0.012	cytokine involved in apoptosis of cancer cells	Predominantly immune cells	immune-related	improved prognosis	-0.00071	1 (1-1)	2.4	0.12	-0.147	0.813
KIR2DL4	1.009	0.048	regulation of NK cell activation	NK cells, T cells	immune-related	improved prognosis	-0.012	0.99 (0.98-1)	9.8	0.0017	1.795	0.000
LAIR2	1.124	0.011	soluble receptor, inhibition of platelet and complement activation	T cells	immune-related	not prognostic	-0.0086	0.99 (0.98-1)	2.6	0.11	0.710	0.074
MMP12	1.345	0.031	degrades soluble and insoluble elastin	Extravillous Trophoblast, Macrophages, B cells	immune-related	not prognostic	-0.000037	1 (1-1)	1.3	0.25	-0.501	0.398
NCF1	0.822	0.033	subunit of NADPH oxidase, mediates neutrophil oxidative burst	Granulocytes, B cells, macrophages	immune-related	not prognostic	-0.0015	1 (1-1)	4.4	0.036	0.694	0.018
NDRG4	-0.691	0.035	cell cycle progression and survival	Astrocytes, endothelial and vascular smooth muscle cells	nonimmune	not prognostic	-0.0000038	1 (1-1)	0	0.94	-0.534	0.130
PDCD1	1.018	0.031	immune checkpoint	T cells	immune-related	improved prognosis	-0.0014	1 (1-1)	4	0.046	1.244	0.000
SH2D2A	0.598	0.012	TCR signal transduction	T cells, NK cells	immune-related	improved prognosis	-0.001	1 (1-1)	8.2	0.0042	0.543	0.006
THEM5	0.637	0.042	involved in fatty acid metabolism	keratinocytes, endothelial cells	nonimmune	improved prognosis	-0.00074	1 (1-1)	1.4	0.24	-0.223	0.677
TIGIT	0.859	0.033	immune checkpoint	T cells	immune-related	improved prognosis	-0.00075	1 (1-1)	5.2	0.023	0.535	0.115
TNFRSF9	0.805	0.035	regulation of survival and development of T cells	T cells, granulocytes, monicytes	immune-related	not prognostic	-0.00038	1 (1-1)	0.83	0.36	0.971	0.002
TYMP	0.744	0.039	promotion of angiogenesis	Myeloid cells	immune-related	not prognostic	-0.000049	1 (1-1)	10	0.0016	0.633	0.030
ZAP70	0.793	0.031	regulation of T cell and B cell activation	T cells	immune-related	improved prognosis	-0.00046	1 (1-1)	3	0.086	0.702	0.017
ZBED2	1.366	0.024	antagonist of interferon regulatory factor 1	Thyroid, T cells, B cells, alveolar type I cells	immune-related	worse prognosis	-0.00015	1 (1-1)	0.9	0.34	1.116	0.013
ZBP1	0.995	0.033	mediates interferon-induced necroptosis	immune cells, melanocytes	immune-related	not prognostic	-0.0009	1 (1-1)	4.8	0.028	0.813	0.018
ZFP57	1.269	0.039	Transcription regulator maintaining maternal and paternal gene imprinting	Brain, muscle	nonimmune	not prognostic	-0.0004	1 (1-1)	0.38	0.54	-0.893	0.292
ADAMDEC1	1.280	0.022	secreted metalloproteinase, immune response regulation	Macrophages, B cells	immune-related	improved prognosis in advanced stage	-0.00028	1 (1-1)	2.3	0.13		
ADGRG5	0.881	0.033	adhesion to GPCR, immune response regulation	Immune cells, alveolar epithelial cells	immune-related	improved prognosis	-0.0027	1 (1-1)	6.9	0.0086		
CHRM3-AS2	0.836	0.035	lncRNA	Predominantly testis	non protein coding	NA	-0.0083	0.99 (0.98-1)	3.1	0.078		
HNRNPA1P21	0.725	0.034	pseudogene	Lymphocytes	non protein coding	NA	-0.004	1 (0.99-1)	3.6	0.059		
KLHDC7B-DT	1.048	0.029	lncRNA	multiple tissues	maybe immune-related	NA	-0.0036	1 (0.99-1)	8.7	0.0032		
LINC00402	0.910	0.032	lncRNA, regulator of allogeneic T-cells	Predominantly immune cells	non protein coding	NA	-0.0037	1 (0.98-1)	0.29	0.59		
SHISAL2A	0.784	0.024	unknown	Macrophages, T cells	immune-related	improved prognosis	-0.0023	1 (0.99-1)	1.6	0.21		
SSTR3	1.046	0.022	somatotsatin receptor, regulation of apoptosis	predominantly testis and brain	testis-associated	improved prognosis	-0.015	0.99 (0.97-1)	3.7	0.054		
TRG-AS1	0.760	0.037	lncRNA, unknown	immune cells	immune-related	NA	-0.0054	0.99 (0.99-1)	6.5	0.011		
TRGC1	0.887	0.033	T cell receptor gamma constant 1	gamma delta T cells	immune-related	NA	-0.02	0.98 (0.97-0.99)	7	0.0083		
TRGC2	0.925	0.044	T cell receptor gamma constant 2	gamma delta T cells	immune-related	NA	-0.0068	0.99 (0.99-1)	7.1	0.0079		
U62317.3	0.948	0.022	lncRNA	unknown	non protein coding	NA	-0.031	0.97 (0.95-0.99)	9.1	0.0026		
