# Supplementary material for: Molecular, Immunological, and Clinical Features Associated With Lymphoid Neogenesis in Muscle Invasive Bladder Cancer
Source: Front Immunol. 2022 Jan 25;12:793992. doi: 10.3389/fimmu.2021.793992 (PMC8821902; doi:10.3389/fimmu.2021.793992)
Supplement: Supplementary file 7 [file DataSheet_7.pdf]

Supplementary Figure legends

Supplementary Figure 1. Schematic representation of the study design
(A) Whorkflow of the TCGA cohort analysis.
(B) Workflow of the IMVigor210 cohort analysis.
(C) Workflow of the retrospective cohort analysis. Colored squares represent regions of interest acquired as multispectral high power fields: tumor tissue was imaged in indicated intratumoral (IT) and peritumoral (PT) areas; all TLS in each slide were imaged irrespective of location.
Yellow boxes represent histological analyses, green boxes – molecular analyses. Curved arrows indicate correlation analyses between corresponding data sets.

Supplementary Figure 2. Schematic representation of the study design
Representative images of FFPE and matched cryosamples of the TCGA MIBC patients with various TLS densities. TLS were defined as intratumoral if were surrounded by tumor cells (white arrowheads) and peritumoral if were present in the adjacent tissue (black arrowheads).
(A) Patient with high TLS density in the diagnostic FFPE section (d-TLS) and also showing TLS in the cryosample (c-TLS).
(B) Patient with high TLS density in the diagnostic FFPE section (d-TLS) and also showing TLS in the cryosample (c-TLS).
(C) Two patients with discrepant histological assessment between the two sampling methods: low d-TLS density but c-TLS-positive.
(D) Peritumoral (PT) and intratumoral (IT) TLS were counted in each diagnostic image, divided by the analysed tissue to obtain the respective densities, and compared by paired t-test.

Supplementary Figure 3. Workflow of quantitative image analysis for the TIL panel
(**A**) FFPE tissue sections were subjected to mIF staining of CD20 (B cells), CD21 (FDCs), CD3 (total T cells), CD8 (cytotoxic T cells), PD-1 (activation marker), TCF1 (marker of naïve cells) and PanCK (tumor cells). Images were acquired by multispectral slide scanner Vectra Polaris. Whole slide scan with marked 200x high power field acquisition areas including all TLS and a representative set of peritumoral (PT) and intratumoral (IT) regions is shown (left). Example of spectrally unmixed high power field image from a region indicated by the red square in the whole slide scan (middle left). Identification of tumor, stroma and TLS tissue segments by trainable tissue segmentation algorithm of the Inform software (middle right). Corresponding cell segmentation map displaying identification of individual cells within each particular tissue segment (right).
(B) Per-cell fluorescence data of all measured dyes was exported and transformed into FlowJo compatible file format. The frequency of various immune cell populations was measured for each tissue category (tumor, stroma, early TLS and mature TLS) in each image. Representative density plots showing overlayed populations of tumor (red) and mature TLS (blue) tissue

categories concatenated form all images is indicated. Parent population title and corresponding daughter population gating frames are indicated in matching colors. Gates were established by verifying the corresponding fluorescent intensities in the Inform cell segmentation tool.

(C) A representative image of a peritumoral region with mature TLS containing $CD21^{+}$ FDC network near a tumor nest ($PanCK^{+}$ cells). Scale abr = 100 μm. Various immune cell populations are depicted in the zoomed regions. Red panel: $CD3^{+}CD8^{+}SH2D2A^{+}PD\text{-}1^{-}TCF1^{-}$ (red arrowhead), $CD3^{+}CD8^{-}PD\text{-}1^{+}TCF1^{-}$ (magenta arrowheads), $CD3^{+}CD8^{-}$ $SH2D2A^{+}PD\text{-}1^{+}TCF1^{+}$ (white arrowheads), $CD20^{+}PD\text{-}1^{-}$ (blue arrowheads). Green panel: $CD3^{+}CD8^{+}SH2D2A^{-}PD\text{-}1^{-}$ $TCF1^{-}$ (orange arrowhead), $CD3^{+}CD8^{+}SH2D2A^{+}PD\text{-}1^{+}TCF1^{-}$ (green arrowhead), $CD3^{+}CD8^{-}$ $SH2D2A^{-}PD\text{-}1^{+}TCF1^{-}$ (cyan arrowhead), $CD20^{+}PD\text{-}1^{+}$ (yellow arrowhead).

Supplementary Figure 4. Survival comparison between patients with no and any number of TLS

Dense lymphocytic aggregates were marked as TLS in all available histology images from diagnostic (FFPE) samples of the TCGA MIBC cohort. Patient groups were defined as having no lymphocytic aggregates (n=80) or any number of such aggregates as assessed in diagnostic images (d-TLS). Overall survival was compared in these groups by Kaplan-Meier curve and log-rank test.

Supplementary Figure 5. Comparison of individual gene and gene expression signature expression in the context of TLS density

(A) Gene expression measured by bulk tumor RNA sequencing of intratumoral regions (cryosamples) was compared in d-TLS density groups by two-tailed t-test. Top four significantly expressed genes are displayed.

(B) Indicated cell population gene signature abundance was determined using the R package Microenvironment Cell Population counter (MCP-counter) and compared between TLS density groups by two-tailed t-test.

(C) Spearman correlation analysis was done to establish the direct relationship between the cell population gene signature abundance and d-TLS density as quartile intervals (TLSq). Color indicates the correlation coefficient value (see scale bar) if significant.

(D) Immune subtype information of the MIBC patients was obtained from Thorsson et al., Immunity, 2018. TLS density was compared in the different immune subtypes by one-way ANOVA.

(E) The proportion of patients with each immune subtypes were compared between TLS density groups.

(F) Overall survival was compared for MIBC patients belonging to the different immune subtypes by Kaplan-Meier curve and log-rank test.

(G) Cell population gene signature abundance was determined using the R package MCP-counter and compared between patients with high or low expression of HRCT1 (top row), ZFP57 (middle row) and NDGR4 (bottom row) by two-tailed t-test.

Supplementary Figure 6. Analysis of neoantigen load in the context of TLS density

(A-D) Spearman correlation analysis was done to establish the direct relationship between the TLS parameters and TMB/neoantigen (NeoAg) count in all TCGA MIBC patients (A), in stage II patients only (B), in stage III patients only (C) and in stage IV patiebts only (D). Color indicates the correlation coefficient value (see scale bar) if significant.

(E-F) TLS density (E) or TMB (F) were compared among different tumor stages by one-way ANOVA.

(G) The count of predicted MHC-binding peptides generated as a result of somatic mutations in each sample was compared between TLS-high and TLS-low tumors by two-tailed t-test.

(H) Patients were split into four groups depending on their TLS density and neoantigen burden using cohort medians as cutoffs for each parameter. Survival was compared by Kaplan-Meier curve and log-rank test.

(I) Cell population gene signature abundance was determined using the R package MCP-counter and compared between TLS-TMB high-high and other patient groups in each tumor stage separately by two-tailed t test with no adjustment for multiple testing.

Supplementary Figure 7. Analysis of tumor immune infiltrates

Characterization of immune cell phenotypes in TME was performed by mIF staining of CD20 (B cells), CD21 (FDCs), CD3 (total T cells), CD8 (cytotoxic T cells), PD-1 (activation marker), TCF1 (marker of naïve cells) and PanCK (tumor cells). Images were acquired by multispectral microscopy from a representative set of peritumoral (PT) and intratumoral (IT) regions. Per-cell fluorescent data were obtained by cell segmentation in tumor nests and stromal regions.

(A) Total $CD8^-$ and $CD8^+$ T cell and B cell frequencies were measured as the proportion of all cells within the respective tissue segments (tumor or stroma). Each data point represents the measured frequency in one tissue segment (image), displayed for every patient and subdivided into TLS density groups and tumor regions. PT, peritumoral; IT – intratumoral.

(B-D) The frequency of indicated cells was measured as the proportion of all cells within the respective tissue segments (tumor or stroma) and averaged per patient in peritumoral and intratumoral regions. The two d-TLS density groups were compared by two-tailed t-test. No correction for multiple testing was done.

Supplementary Figure 8. Analysis of tumor immune clusters

(A) Frequencies of identified tumor immune clusters (TIL composition) were assessed as the proportion of all analyzed tissue segments (tumor or stroma) in each patient. NI, non-infilrtated; CD8hi, $CD8^+$ T cell infiltrated, high frequency; CD8lo, $CD8^+$ T cell infiltrated, low frequency; BT, B and $CD8^+$ T cell co-infiltrated; Bcells, predominant B cell infiltration.

(B) Frequency of indicated populations was assessed as the proportion of total cells (top row) or as the proportion of the parent population (bottom row) measured in the indicated tissue segment in each image. Frequencies were compared in the identified tumor immune clusters by one-way ANOVA.

(C) Proportion of PD-1^+TCF1^+ progenitor-like cells from total $CD8^-$ (top) and $CD8^+$ (bottom) T cells was measured in each individual tissue segment (image) and compared in different stromal immune clusters for TLS-high and TLS-low tumors separately using two-tailed t-test and one-way ANOVA. No correction for multiple testing was done.

Supplementary Figure 9. Analysis of TLS composition

Characterization of immune cell infiltration in TLS was performed by mIF staining as described in Supplementary Figure . Presence of CD21+ cells was used to define a TLS as mature or immature.

(A) Frequencies of B cells and T cells were averaged per patient and compared between TLS maturation stages separately in each TLS density group by two-tailed t-test.

(B) Frequencies of B cells and T cells were averaged per patient and compared between TLS density groups for each maturation stage separately by two-tailed t-test.

(C) Proportions of the indicated populations were averaged per patient and compared between TLS maturation stages for TLS-high and TLS-low patients separately by two-tailed t-test.

(D) Frequencies of identified TLS subtypes were assessed as the proportion of all analyzed TLS in each patient. ImmCD8hi, immature TLS with high $CD8^+$ T cell infiltration; ImmCD8lo, immature TLS with low $CD8^+$ T cell infiltration; MatCD8hi, mature TLS with high $CD8^+$ T cell infiltration; MatCD8lo, mature TLS with low $CD8^+$ T cell infiltration.

(E) Frequency of each indicated TLS subtype was compared between TLS density groups by two-tailed t-test.

(F) Frequency of indicated populations was assessed as the proportion of the parent population measured in each TLS. Frequencies were compared in the identified TLS subtypes by one-way ANOVA in separately for the two TLS density groups.

(G) Frequency of the indicated population was assessed as the proportion of the parent population measured in each TLS and averaged in each patient by TLS subtype. Frequencies were compared between TLS density groups for each TSL subtype separately by two-tailed t-test.
