# Supplementary figures and images for: Molecular, Immunological, and Clinical Features Associated With Lymphoid Neogenesis in Muscle Invasive Bladder Cancer
Source: Front Immunol. 2022 Jan 25;12:793992. doi: 10.3389/fimmu.2021.793992 (PMC8821902; doi:10.3389/fimmu.2021.793992)

# Supplementary Figure 1

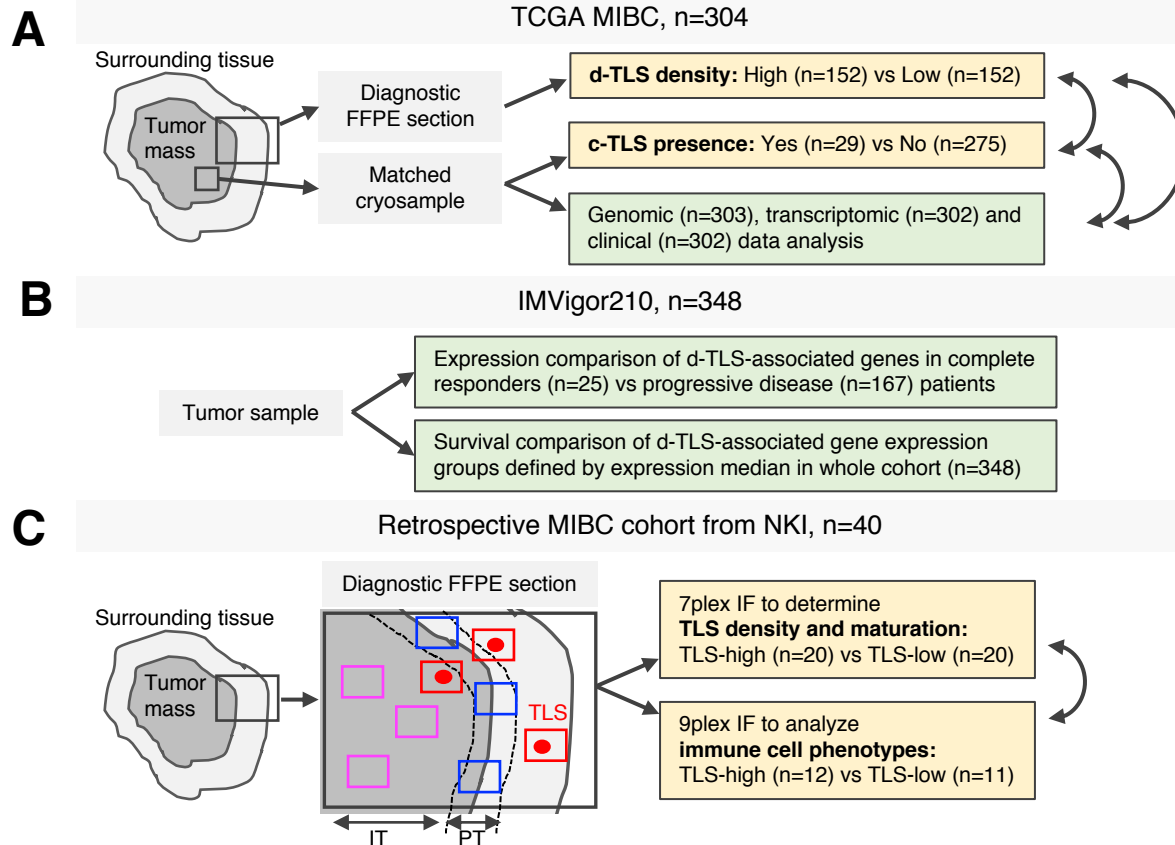

Supplement: Supplementary file 8 [file DataSheet_8.pdf]

## Supplementary Figure 2

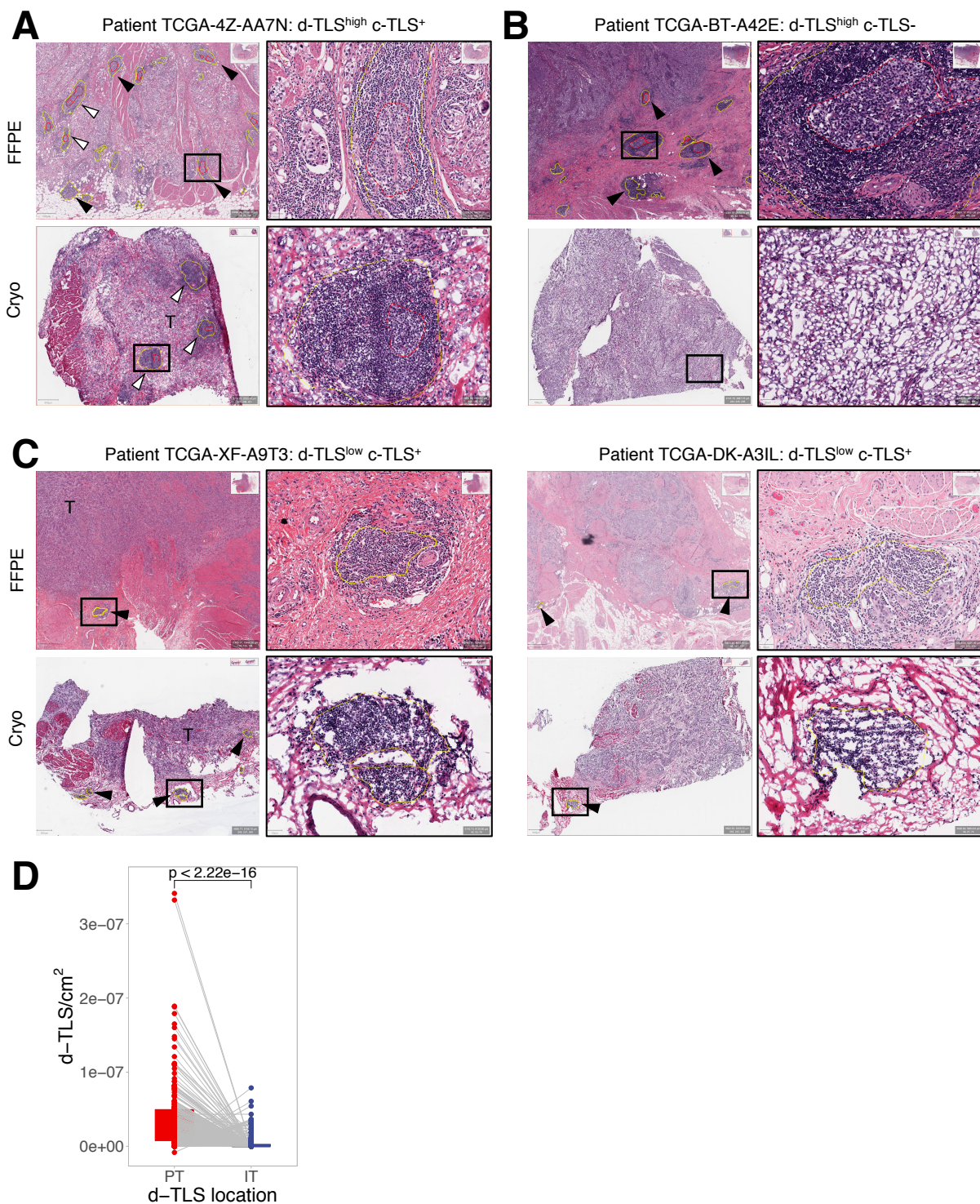

Supplement: Supplementary file 9 [file DataSheet_9.pdf]

# Supplementary Figure 3

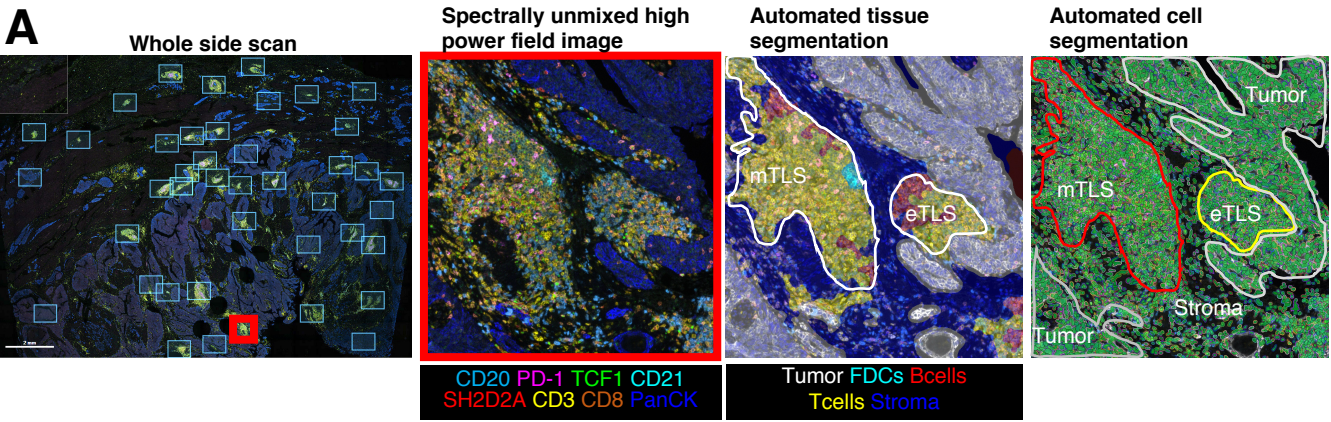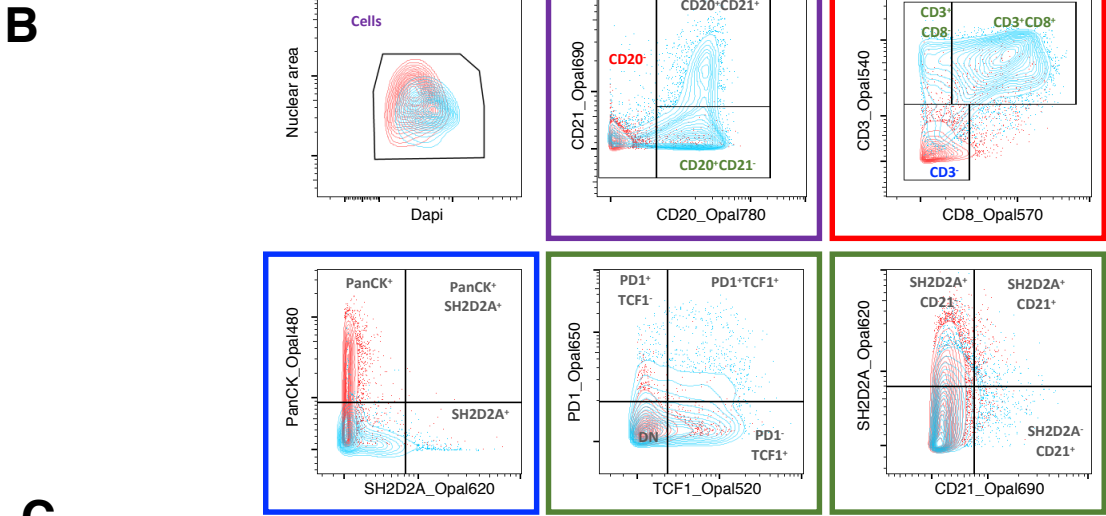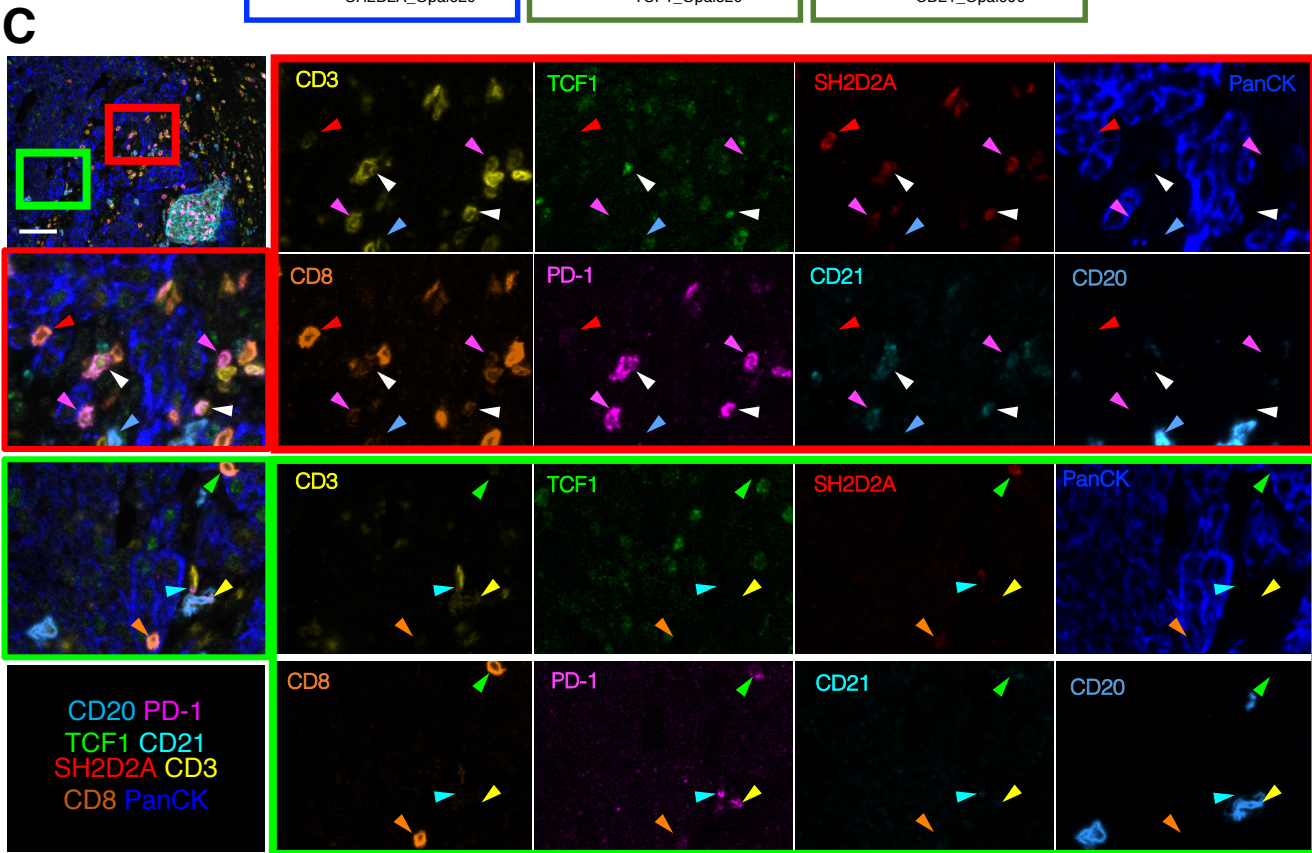

Supplement: Supplementary file 10 [file DataSheet_10.pdf]

# Supplementary Figure 4

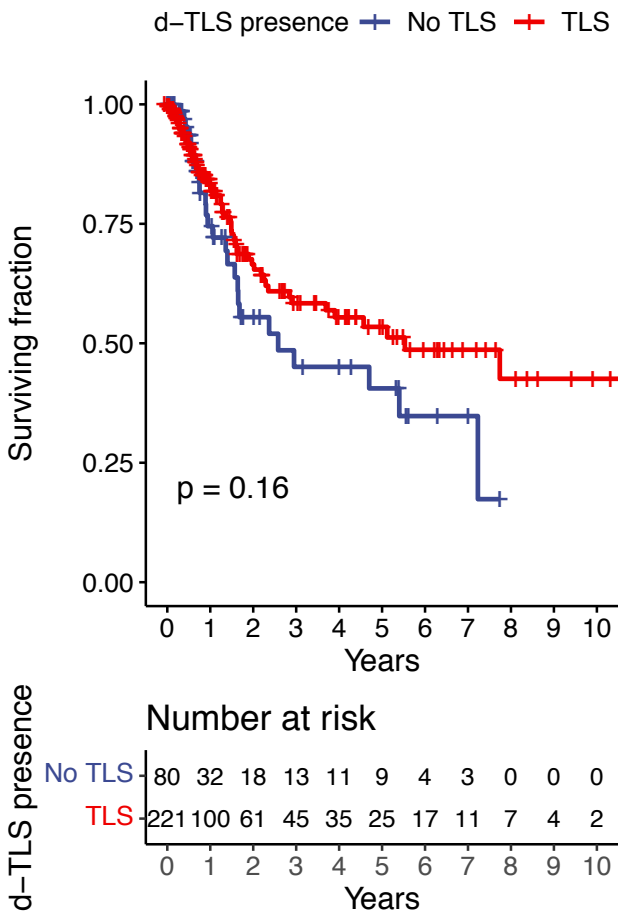

Supplement: Supplementary file 11 [file DataSheet_11.pdf]

Supplementary Figure 5

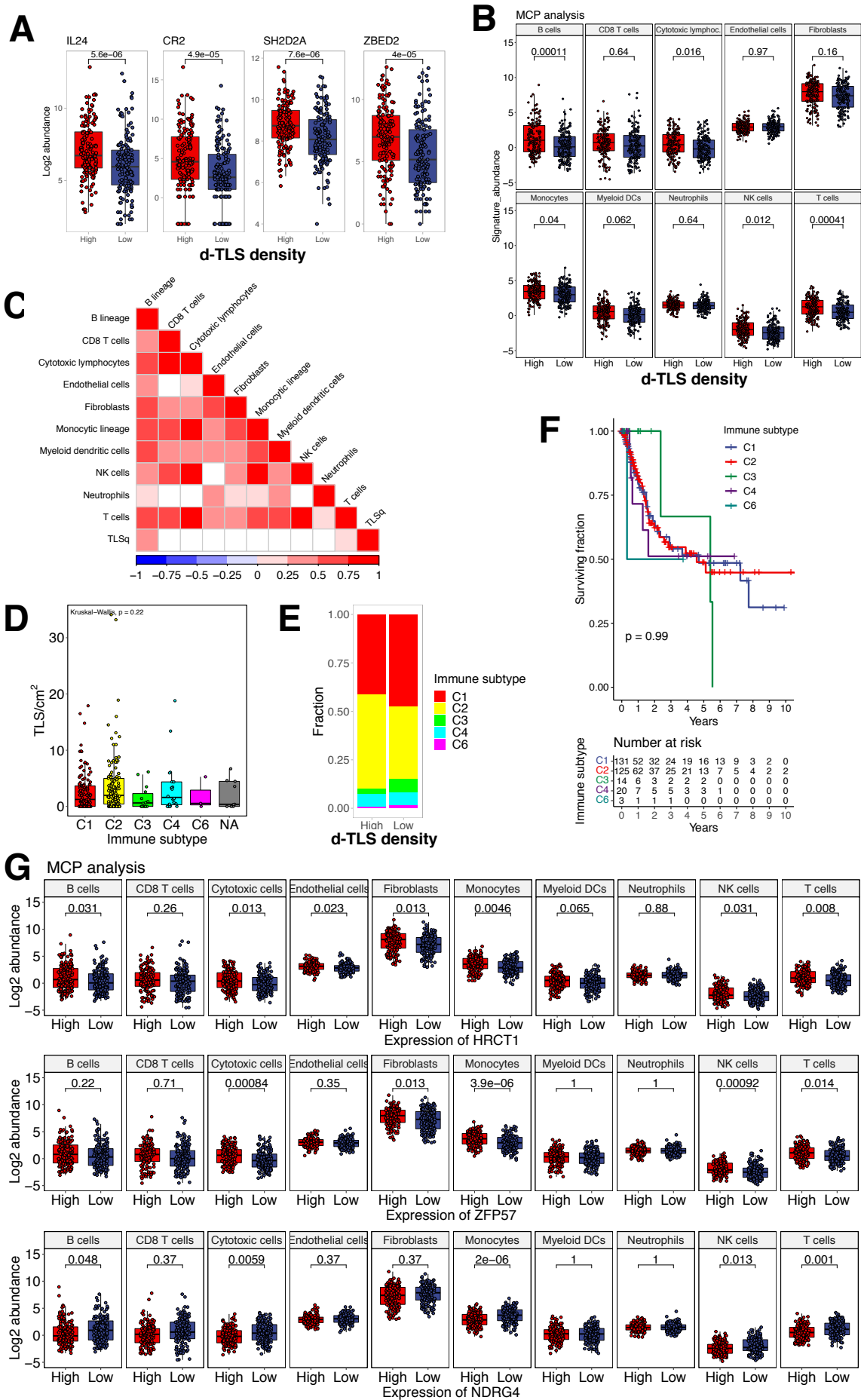

Supplement: Supplementary file 12 [file DataSheet_12.pdf]

Supplementary Figure 6

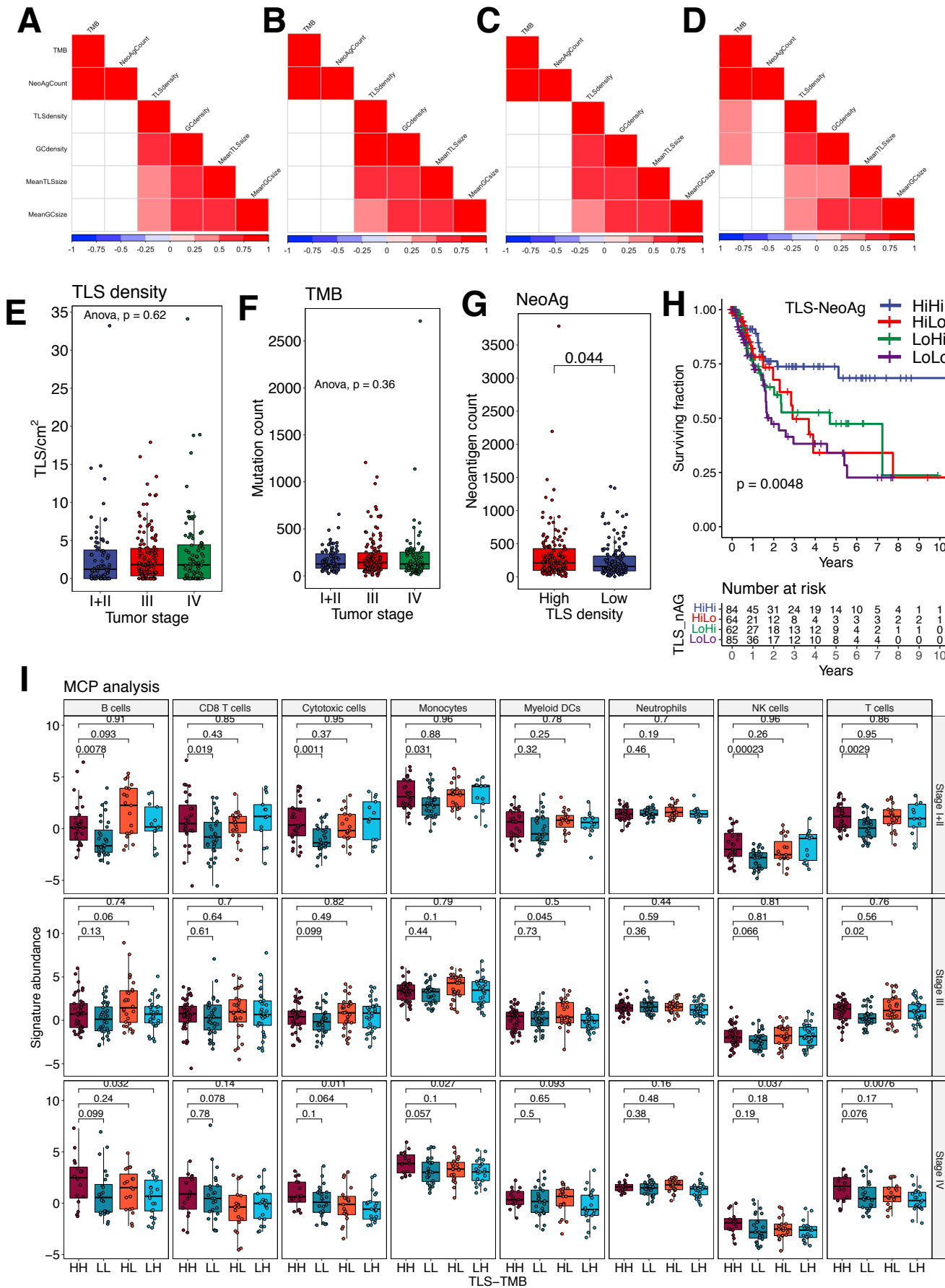

Supplement: Supplementary file 13 [file DataSheet_13.pdf]

# Supplementary Figure 7

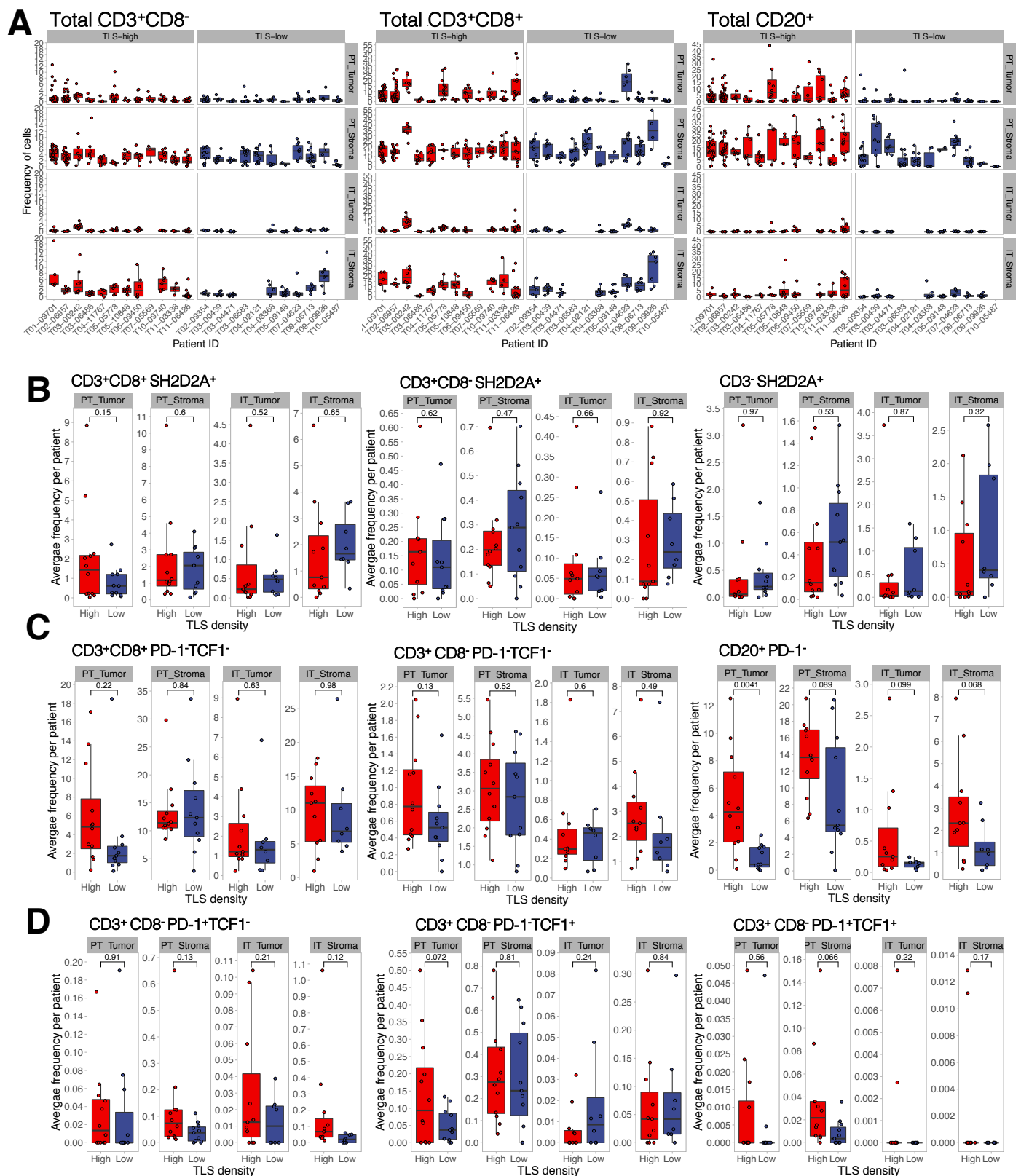

Supplement: Supplementary file 14 [file DataSheet_14.pdf]

# Supplementary Figure 8

**A**

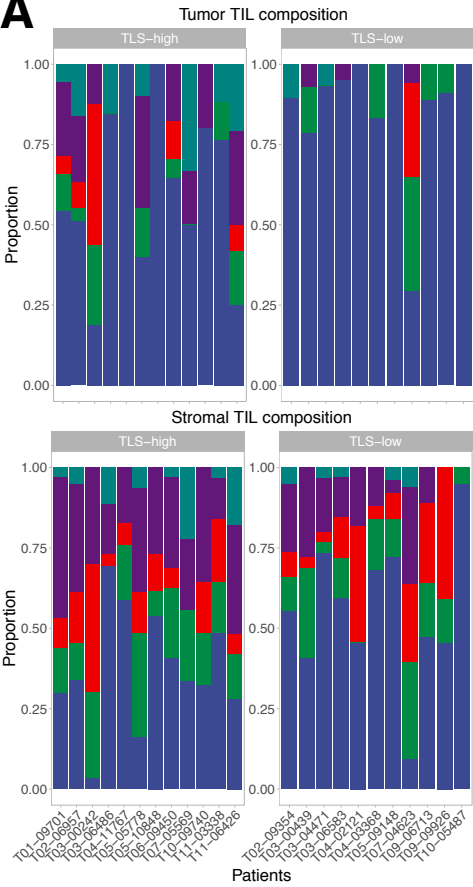

**Immune clusters**

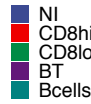

**B**

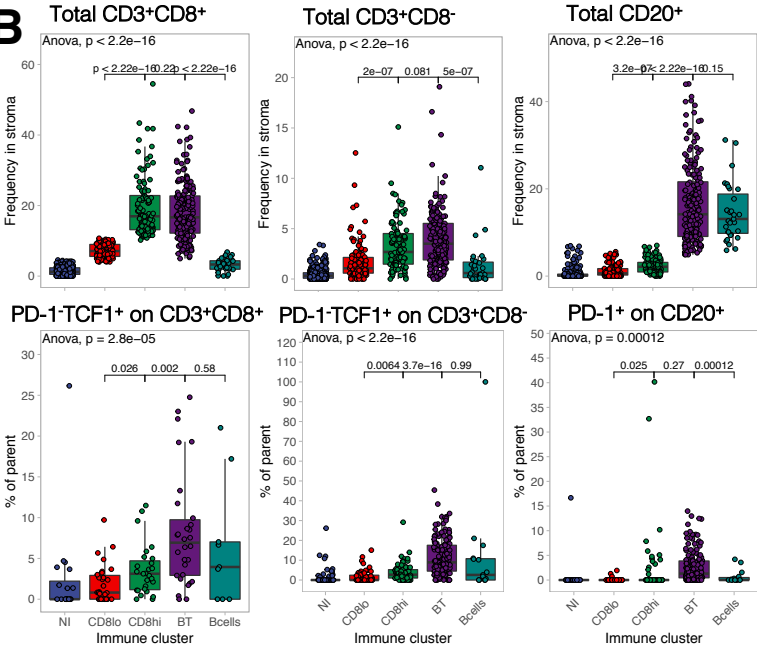

**C**

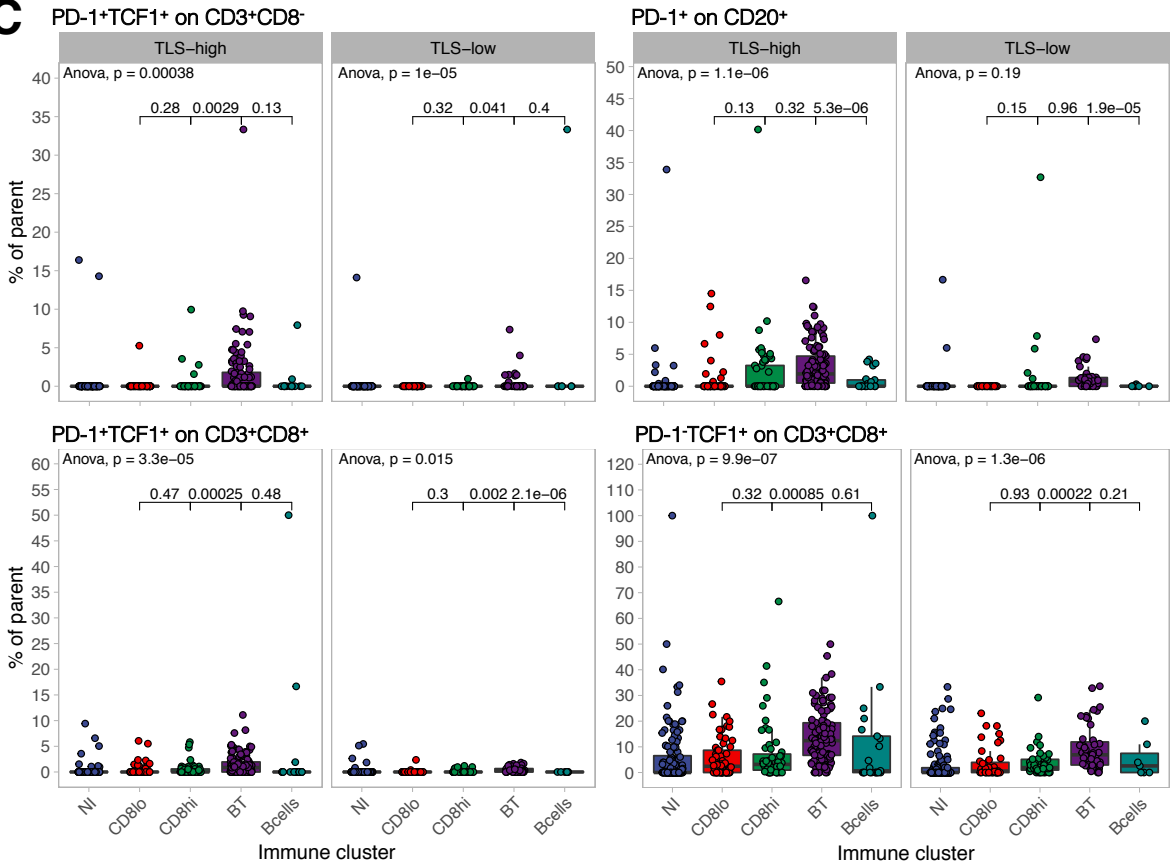

Supplement: Supplementary file 15 [file DataSheet_15.pdf]
